# Pterin-based small molecule inhibitor capable of binding to the secondary pocket in the active site of ricin-toxin A chain

**DOI:** 10.1371/journal.pone.0277770

**Published:** 2022-12-12

**Authors:** Ryota Saito, Masaru Goto, Shun Katakura, Taro Ohba, Rena Kawata, Kazuki Nagatsu, Shoko Higashi, Kaede Kurisu, Kaori Matsumoto, Kouta Ohtsuka

**Affiliations:** 1 Department of Chemistry, Faculty of Science, Toho University, Funabashi, Chiba, Japan; 2 Research Center for Materials with Integrated Properties, Toho University, Funabashi, Chiba, Japan; 3 Department of Molecular Bioscience, Faculty of Science, Toho University, Funabashi, Chiba, Japan; Weizmann Institute of Science, ISRAEL

## Abstract

The Ricin toxin A chain (RTA), which depurinates an adenine base at a specific region of the ribosome leading to death, has two adjacent specificity pockets in its active site. Based on this structural information, many attempts have been made to develop small-molecule RTA inhibitors that simultaneously block the two pockets. However, no attempt has been successful. In the present study, we synthesized pterin-7-carboxamides with tripeptide pendants and found that one of them interacts with both pockets simultaneously to exhibit good RTA inhibitory activity. X-ray crystallographic analysis of the RTA crystal with the new inhibitor revealed that the conformational change of Tyr80 is an important factor that allows the inhibitors to plug the two pockets simultaneously.

## Introduction

**Fig 1:**
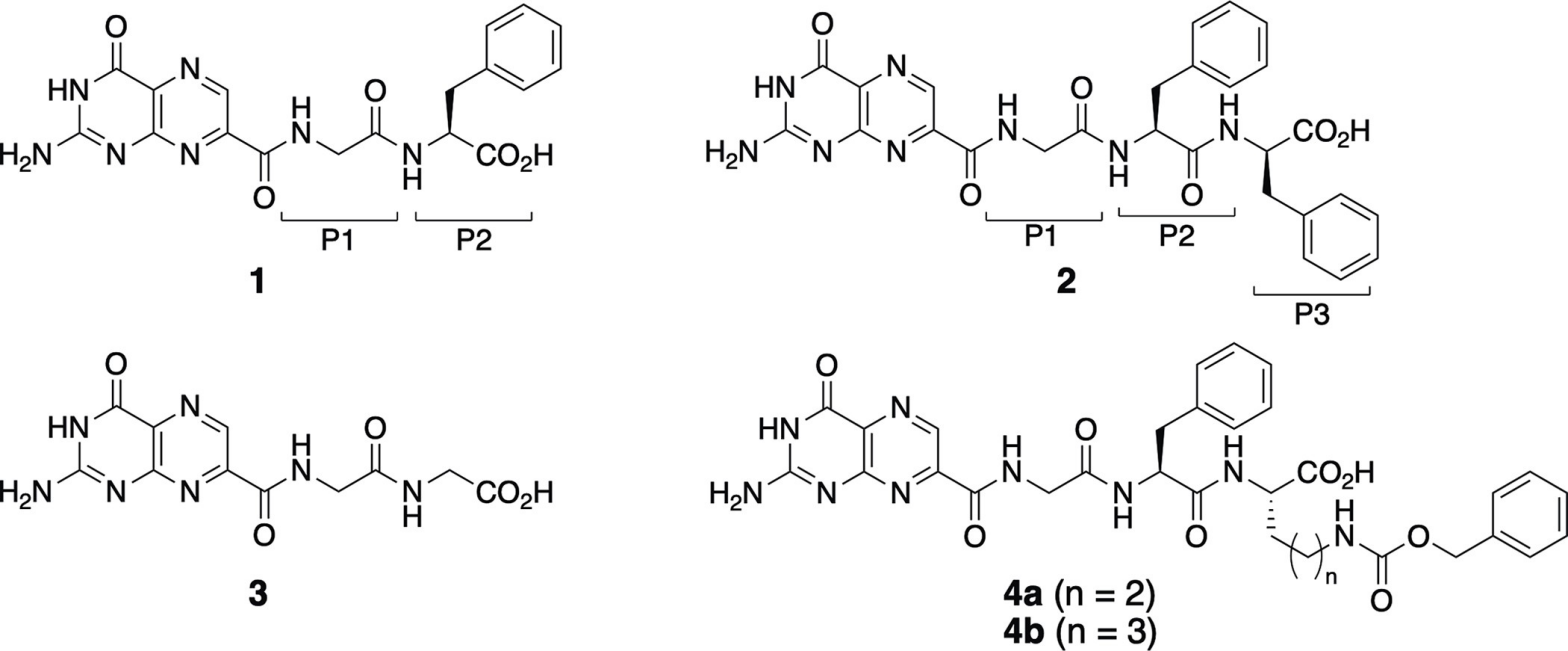
Structures of pterin-7-carboxamides 1–4.

Ricin is a highly toxic protein that can be easily isolated from the seeds of *Ricinus communis*; however, no effective antidote has been developed against it [1]. Ricin is an AB toxin consisting of an enzymatically active A subunit (Ricin Toxin A-chain, RTA) and a cell-binding B subunit (Ricin Toxin B-chain, RTB) linked by a disulfide bond [2, 3]. RTA is highly catalytically active in depurinating the adenine base at position 4324 (A_4324_) selectively in a specific region in the 28S rRNA to inhibit ribosomal protein biosynthesis, causing cell death [4]. The active site of RTA consists of two pockets, the primary pocket catalyzing the depurination of A_4324_ and the secondary pocket accommodating the guanine residue adjacent to A_4324_, with Tyr80 dividing these pockets. Based on the structural information of RTA, many inhibitor candidates based on nucleobases, pterins, and pyrimidines have been proposed, but all have been found to exhibit low RTA inhibitory activity [1]. Subsequently, Schramm *et al*. reported that the cyclic transition state model showed high RTA inhibitory activity, with an IC_50_ of 1 μM [5]. X-ray crystallographic analysis of this compound complexed with RTA showed that the model compound interacted with both the primary and secondary pockets. Since all the RTA inhibitors reported so far interacted only with the primary pocket, this result suggests that simultaneous interaction with the two pockets is a key factor for high RTA inhibitory activity. Despite its fascinating inhibitory activity, the cyclic model compound has not been put to practical use as an antidote for RTA because its high inhibitory activity was measured under non-physiological conditions and its synthesis is complex. Therefore, there is a requirement for development of more easily accessible and small-molecule RTA inhibitors. Our research group has also been interested in developing RTA inhibitors and has reported that several pterin-7-carboxamides (7PCAs) exhibited weak to good inhibition of RTA *in vitro* [6]. For instance, 7PCAs with (glycyl)phenylalanine pendants (**1** and **2** in Fig 1) exhibited high RTA inhibitory activity (IC_50_ = 20 and 15 μM, respectively), while the corresponding glycylglycine analog (**3**) was found to be a weak RTA inhibitor (IC_50_ = 400 μM). X-ray crystallographic analysis of **2**/RTA complex revealed that the C-terminal residue of the tripeptide pendant (P3) was oriented toward the secondary pocket, but was not long enough to reach it [6]. This indicates that a 7PCA with a tripeptide pendant with an appropriately modified the C-terminal residue can interact simultaneously with the primary and secondary pockets. Thus, in this study, we report the synthesis of 7PCAs with tripeptide pendants having elongated C-terminal residues, in which ornithine and lysine residues with an aromatic ring were employed as the C-terminal residues to direct the interaction with the secondary pocket. X-ray crystallographic analysis of the new ligands revealed that they complexed with RTA to be the first example of a small-molecule inhibitor that binds simultaneously with the primary and the secondary pockets of RTA.

## Results and discussion

**Scheme 1:**
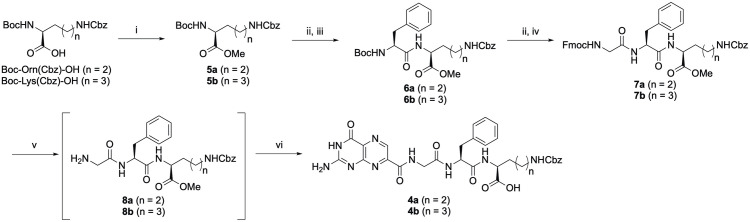
Synthesis of compounds 4a and 4b. *Reagents and conditions*: (i) CH_3_I, K_2_CO_3_, dimethylformamide (DMF), 0°C to r.t.; (ii) 4-M HCl in 1,4-dioxane, r.t.; (iii) Boc-Phe-OH, PyBOP, ^*i*^Pr_2_NEt, DMF, r.t.; (iv) Fmoc-Gly-OH, PyBOP, ^*i*^Pr_2_NEt, DMF, r.t.; (v) piperidine, DMF, r.t.; (vi) 7-methoxycarbonylpterin, DBU, MeOH, r.t.

The synthesis of new 7PCAs, **4a** and **4b**, were started from appropriate commercially available amino acid derivatives, which were converted into the corresponding methyl esters **5a** and **5b**. **5a** and **5b** were then processed using the conventional peptide synthetic procedures to produce crude tripeptides **8a** and **8b**, respectively. Finally, the tripeptides were respectively reacted with 7-methoxycarbonylpterin in the presence of 1,8-diazabicyclo [5.4.0]undec-7-ene (DBU) as an accererator [6–9] to synthesize the corresponding 7PCAs, **4a** and **4b** (Scheme 1).

**Fig 2:**
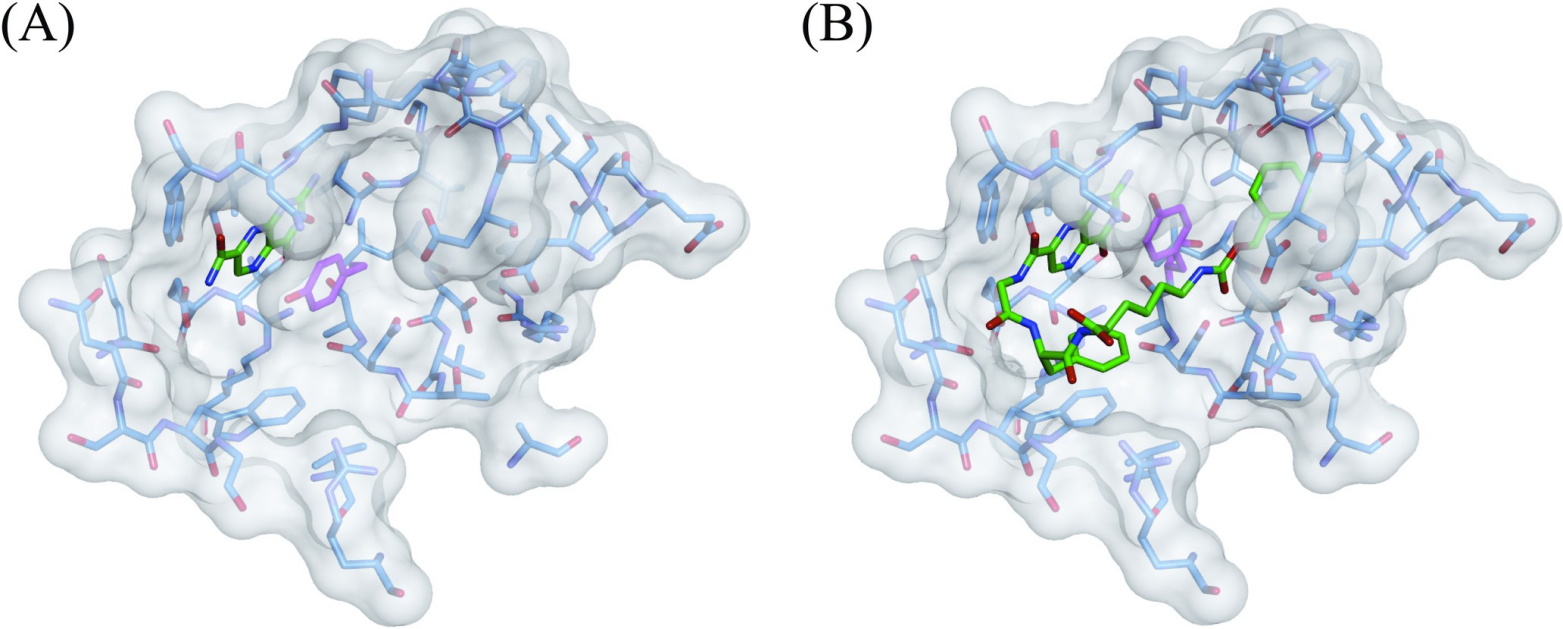
X-ray crystal structures of (A) 4a-RTA and (B) 4b-RTA complexes in surface representation styles. The ligands are colored in green, and the amino acid residues around the ligands are in blue except Tyr80 in magenta.

**Fig 3:**
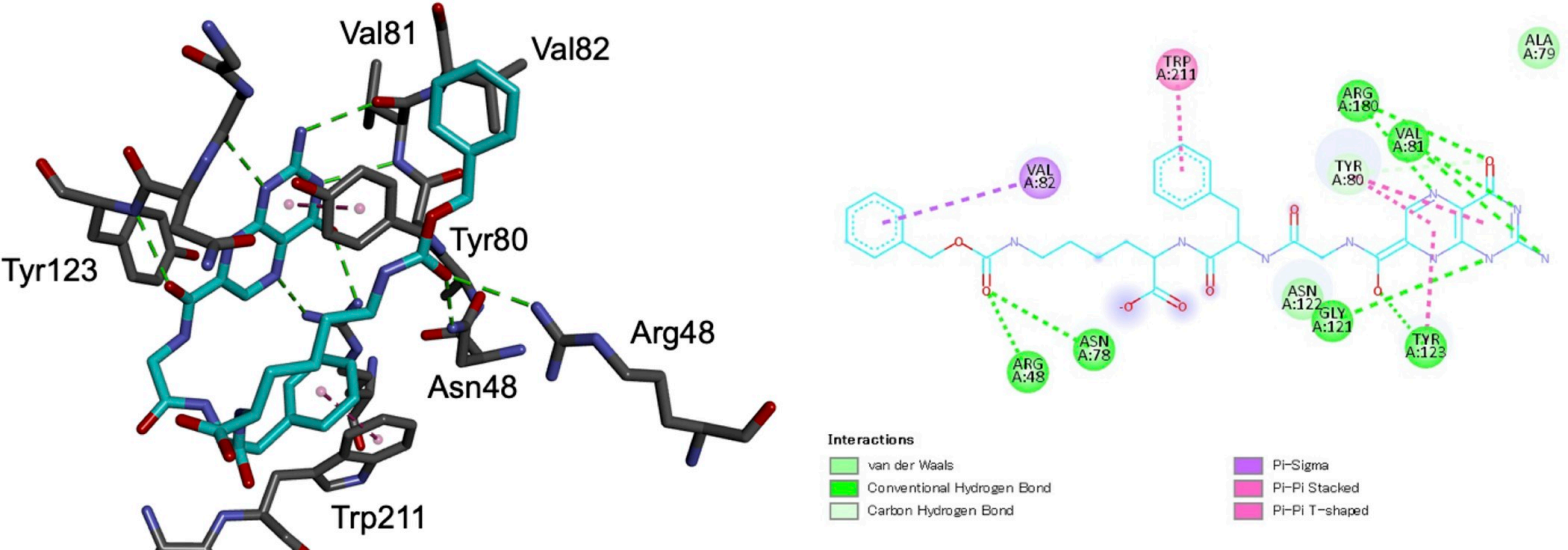
Interactions between 4b and RTA. All interactions were detected and visualized by Discovery Studio Visualizer [11] in both 2D (right) and 3D (left) styles.

Co-crystals of RTA with **4a** and **4b** were prepared using the hanging drop vapor diffusion crystallization method and their crystal structures were analyzed. The resulting 3D structures of the complexes are shown in Fig 2. In each case, the pterin ring interacted with Gly121, Asn122, Tyr123, Ile172, and Arg180 in the primary pocket of RTA through the same interactions as those previously observed with other pterin derivatives [6–8, 10]. This demonstrates that the binding mode of the pterin moiety of 7PCAs to the primary pocket of RTA is constant, regardless of the structure of the pendants. For **4a**, the tripeptide moiety was unsuccessfully assigned, probably because only the pterin moiety was accommodated in the primary pocket and the pendant moiety was free to move. In stark contrast, the entire structure of **4b** was assigned, and, notably, the Cbz group on the C-terminal residue was accommodated in the secondary pocket. This is the first example of a single and small organic molecule plugging the two pockets in the active site of RTA simultaneously. Closer examination of the crystal structure of **4b**/RTA complex revealed that the phenylalanine residue in **4b** interacts with Trp211 of RTA in a T-shaped CH–π interaction, and Tyr80, the partition between the two pockets in the active site of RTA, was moved to the opposite side of Trp211 owing to repulsion with the phenyl group. Besides, the carbonyl group of the Cbz in **4b** made hydrogen bonds with Asn48 and Arg78 in the “entrance” of the secondary pocket at 3.15 Å and 2.67 Å, respectively, and accordingly the benzene ring of Cbz group was placed in the secondary pocket (Fig 3).

**Fig 4:**
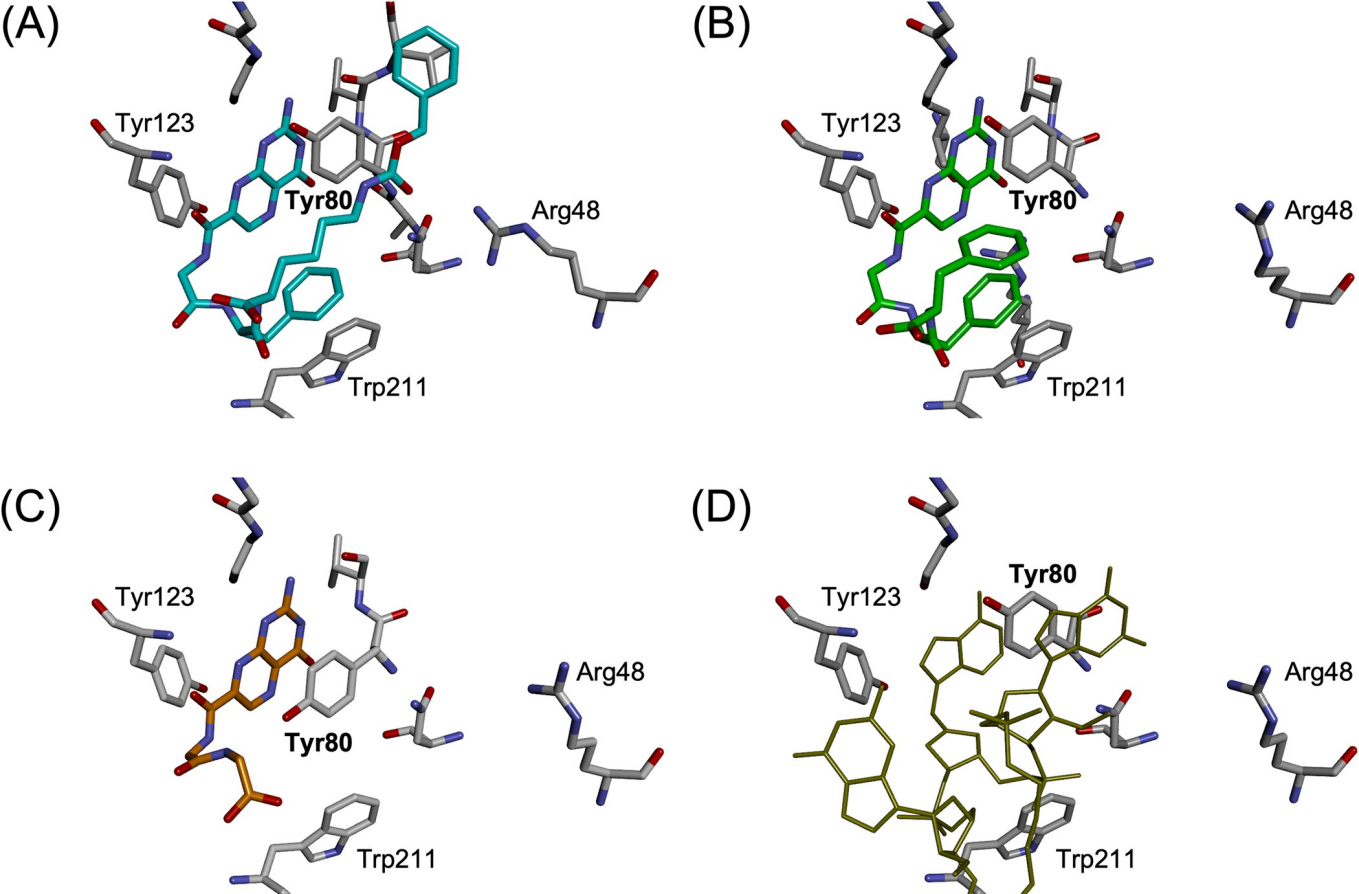
Comparison of the crystal structures of RTA-inhibitor complexes. (A) crystal structure of **4b**-RTA complex, (B) crystal structure of **2**-RTA complex (PDB ID: 4HUP), (C) crystal structure of **3**-RTA complex (PDB ID: 4HV7), and (D) crystal structure of RTAS complexed with the cyclic transition-model inhibitor (PDB ID: 3HIO). Images are drawn by Discovery Studio Visualizer [11].

The same movements of Tyr80 were observed in the crystal structures of RTA with the potent inhibitors **1**, **2** (Fig 4B) [6], while no such conformational change was observed in RTA crystals with **4a** and other low-activity inhibitors such as **3** (Fig 4C), for which the positions of Tyr80 was the same as in the inhibitor-free crystal structure [6]. Furthermore, the same rearrangement of Tyr80 residue was observed in the crystal structure of RTA with the Schramm’s cyclic nucleotide as a ligand (Fig 4D). These results strongly suggest that the conformational change in Tyr80 is essential for inhibitors to interact with the secondary pocket and to increase the inhibitory activity. For 4A, the displacement of Tyr80, as well as the T-shaped interactions, were not observed, even though it had the same P1-P2 moiety, Gly-Phe. It is likely that the alkyl chain of the C-terminal residue in 4A is not long enough to form the hydrogen bonds with the residues at the entrance of the secondary pocket; consequently, the unfixed large side chain is free to move, preventing the phenyl ring in P2 from forming a T-shape interaction.

The activity of RTA in the absence and presence of **4a** and **4b** was measured using an *in vitro* translation reaction of firefly luciferase utilizing rabbit reticulocyte lysate and a subsequent luciferase quantification by measuring the bioluminescence intensity. The inhibitory activity of **1**, as a positive standard, was also evaluated. The results are presented in Table 1.

**Table 1:** RTA inhibitory activity of 1, 4a, and 4b.

Compound	IC_50_ /μM
**1**	30.8 ± 9.1
**4a**	33.4 ± 2.7
**4b**	485 ± 33

Compound **4b** exhibited an IC_50_ value of 33.4±2.7 μM, whereas compound **4a** exhibited an IC_50_ value of 485±33 μM. An approximately 10-fold difference was observed between the two inhibitory activities. The weak inhibitory activity of **4a** was comparable to that of pterin-7-carboxylic acid, which is consistent with the fact that only the pterin-7-carboxylic acid portion was assigned in the X-ray crystallography of **4a**/RTA and no conformational change in Tyr80 was observed. The more potent **4b**, which changed the orientation of Tyr80, exhibited the same level of inhibitory activity as **1** and **2**, bringing about the same movement of Tyr80. From this result, it appears that the inhibitory activity was not enhanced by the inhibitor plugging the secondary pocket. This apparent ineffectiveness may be due to the fact that the Cbz group is located at a shallow position near the entrance of the secondary pocket. Therefore, replacement of the Cbz group with larger aromatic ring systems having substituents of forming extra interactions with residues in the secondary pocket could lead to the enhancement of the RTA inhibitory activity. More detailed structure-activity relationship studies to determine the residues in the secondary pocket to be interactes with will be conducted in the near future.

## Conclusion

In the present study, new pterin compounds **4a** and **4b** were synthesized; among them, **4b**, having a Gly-Phe-Lys(Cbz)-OH pendant, exhibited good RTA inhibitory activity comparable to **1** and **2** previously reported as potent RTA inhibitors. X-ray crystallographic analysis of **4b**/RTA co-crystal revealed that the potent **4b** not only bound tightly to the primary pocket but also plugged the secondary pocket in the active site of RTA. This is the first example of a small organic inhibitor that is capable of simultaneously plugging the two pockets of the RTA sensitive site. A more detailed analysis of **4b**/RTA co-crystal revealed that the conformational change in Tyr80, induced by the T-shaped interaction between the phenyl ring in the pendant moiety of **4b** and Trp211, is an important factor in determining the RTA inhibitory activity and achieving the interaction between inhibitors and the secondary pocket of the RTA active site. These findings should guide the development of more potent RTA inhibitors.

## Materials and methods

### General

^1^H-NMR spectra were recorded using an ECP-400 spectrometer (JEOL Ltd., Japan). Chemical shifts (*δ*) are reported in ppm using tetramethylsilane or an undeuterated solvent as the internal standard in the deuterated solvent used. The coupling constants (*J*) are given in hertz. ^13^C-NMR spectra were recorded using an ECP-100 spectrometer (JEOL Ltd., Japan) or an Avance II 400 spectrometer (Bruker Biospin, Billerica, MA, USA). Chemical shift multiplicities are reported as s = singlet, d = doublet, t = triplet, and m = multiplet. Electrospray ionization (ESI) mass spectra were recorded on a JEOL JMS-T100CS mass spectrometer. Column chromatography was performed on silica gel (particle size: 46–50 μm; Fuji Silysia Chemical Ltd.). Sephadex^TM^ LH-20, purchased from Cytiva (Tokyo, Japan), was used for the gel filtration. All chemicals were purchased from Sigma-Aldrich Co. LLC. or Tokyo Chemical Industry Co., Ltd. (TCI) and used as received, unless otherwise mentioned below.

### Chemistry

#### Synthesis of Boc-Orn(Cbz)-OMe (5a) [12]

CH_3_I (500 μL, 8 mmol) was added dropwise to a suspension of Boc-Orn(Cbz)-OH (1.84 g, 5 mmol) and K_2_CO_3_ (1.43 g, 10 mmol) in DMF (10 mL) at 0°C, and the mixture was stirred at 0°C for 30 min, then room temperature for overnight. The mixture was then diluted with ethyl acetate, washed with water followed by brine, and dried over anhydrous Na_2_SO_4_. Evaporation of the solvent resulted in the formation of **5a** as a colorless oil (1.86 g, quant.). ^1^H NMR (400 MHz, CDCl_3_): *δ*/ppm 1.55 (9H, s), 1.57–1.67 (3H, m), 1.81–1.87 (1H, m), 3.22 (2H, dt, *J* = 12.8, 6.6 Hz), 3.73 (3H, s), 4.30 (1H, dt, *J* = 12.8, 6.6 Hz), 4.83 (1H, br s), 5.05 (1H, br s), 5.09 (2H, s), 7.36 (5H, s). ^13^C NMR (100 MHz, CDCl_3_): *δ*/ppm 26.02, 28.37, 30.09, 40.56, 50.77, 52.44, 53.14, 66.72, 80.08, 128.17, 128.58, 136.60, 155.51, 156.54, 173.16. HR-ESIMS Calcd for C_19_H_28_N_2_NaO_6_ [M+Na]^+^ 403.1845. Found 403.1853.

#### Synthesis of Boc-Lys(Cbz)-OMe (5b)

CH_3_I (600 μL, 9.6 mmol) was added dropwise to a suspension of Boc-Lys(Cbz)-OH (2.28 g, 6 mmol) and K_2_CO_3_ (1.67 g, 12 mmol) in DMF (10 mL) at 0°C, and the mixture was stirred at 0°C for 30 min, and then at room temperature overnight. The mixture was then diluted with ethyl acetate, washed with water followed by brine, and dried over anhydrous Na_2_SO_4_. Evaporation of the solvent resulted in the formation of **5b** as a colorless oil (2.28 g, quant.). ^1^H NMR (400 MHz, CDCl_3_): *δ*/ppm 1.34–1.40 (2H, m), 1.43 (9H, s), 1.43–1.54 (2H, m), 1.61–1.65 (1H, m), 1.74–1.90 (1H, m), 3.19 (2H, dt, *J* = 13.0, 6.0 Hz), 3.73 (3H, s), 4.29 (1H, dd, *J* = 8.4, 12.3 Hz), 4.79 (1H, br s), 5.05 (1H, br s), 5.09 (2H, s), 7.35 (5H, s). ^13^C NMR (100 MHz, CDCl_3_): *δ*/ppm 22.51, 28.44, 29.49, 32.51, 40.75, 52.43, 53.27, 66.78, 80.07, 128.23, 128.64, 136.70, 155.60, 156.58, 173.39. These spectral data are congruent with those previously reported [13, 14].

#### Synthesis of H-Orn(Cbz)-OMe hydrochloride

To a solution of **5a** (1.22 g, 3 mmol) in 1,4-dioxane (10 mL), 4 M HCl in 1,4-dioxane (10 mL) was added at room temperature, and the mixture was stirred at room temperature for 8 h. After the completion of the reaction, HCl and 1,4-dioxane were removed by evaporation. The residual solvents were further removed by azeotropy with benzene three times to give H-Orn(Cbz)-OMe hydrochloride as a colorless solid (1.02 g, quant.). ^1^H NMR (400 MHz, CDCl_3_): *δ*/ppm 1.47–1.81 (2H, m), 1.89–2.17 (2H, m), 3.01–3.22 (2H, m), 3.71 (3H, s), 4.00–4.17 (1H, m), 5.04 (1H, s), 5.68 (1H, br s), 7.27–7.32 (5H, m), 8.63 (3H, br s); ^13^C NMR (100 MHz, CDCl_3_): *δ*/ppm 25.39, 27.58, 40.24, 53.05, 53.58, 66.61, 128.09, 128.56, 128.74, 136.75, 156.86, 188.43. These spectral data are congruent with those previously reported [12].

#### Synthesis of Boc-Phe-Orn(Cbz)-OMe (6a)

H-Orn(Cbz)-OMe hydrocloride (1.60 g, 5.1 mmol) was dissolved in DMF (20 mL), and Boc-Phe-OH (1.52 g, 5.7 mmol), PyBOP (3.33 g, 6.4 mmol) and diisopropylethylamine (1.90 mL, 11.0 mmol) were successively added to the DMF solution. The mixture was stirred for 2 days at room temperature. The reaction mixture was poured into H_2_O (50 mL), and the organic materials were extracted with ethyl acetate (50 mL). The organic layer was successively washed with 1M KHSO_4_ (150 mL), saturated Na_2_CO_3_ aq. (150 mL), and brine (150 mL), and then dried over anhydrous Na_2_SO_4_. After removal of the solvent under reduced pressure, the crude product was purified by column chromatography on silica gel (140 g) and eluted with hexane/ethyl acetate = 1/2 to give **6a** as a colorless solid (2.04 g, 76%). ^1^H NMR (400 MHz, CDCl_3_): *δ*/ppm 1.40 (9H, s), 1.44–1.67 (3H, m), 1.80–1.86 (1H, m), 3.06 (2H, d, *J* = 6.7 Hz), 3.17 (2H, dt, *J* = 13.5, 6.7 Hz), 3.69 (3H, s), 4.35 (1H, d, *J* = 6.7 Hz), 4.54 (1H, dd, *J* = 7.8, 13.5 Hz), 4.91 (1H, br s), 5.03 (1H, br s), 5.09 (2H, s), 6.53 (1H, d, *J* = 7.8 Hz), 7.18–7.24 (5H, m), 7.28–7.36 (5H, m). ^13^C NMR (100 MHz, CDCl_3_): *δ*/ppm 25.87, 28.39, 29.67, 38.24, 40.51, 52.00, 52.60, 56.03, 66.84, 80.45, 127.11, 128.25(×2), 128.66, 128.80, 129.49, 136.65, 136.71, 156.60(×2), 171.22, 172.13. HR-ESIMS Calcd for C_28_H_37_N_3_NaO_7_ [M+Na]^+^ 550.2529. Found 550.2507.

#### Synthesis of H-Lys(Cbz)-OMe hydrochloride

To a solution of **5b** (1.57 g, 4.7 mmol) in 1,4-dioxane (10 mL) 4 M HCl in 1,4-dioxane (10mL) was added at room temperature, and the mixture was stirred at room temperature for 3 h. After the completion of the reaction, HCl and 1,4-dioxane were removed by evaporation. Residual solvents were further removed by azeotropy with benzene three times to give H-Lys(Cbz)-OMe hydrochloride [14, 15] as colorless solid (1.30 g, quant.). ^1^H NMR (400 MHz, CDCl_3_): *δ*/ppm 1.36–1.71 (4H, m), 1.90–2.11 (2H, m), 3.07–3.22 (2H, m), 3.75 (3H, s), 4.00–4.23 (1H, m), 5.06 (2H, s), 5.50 (1H, br s), 7.27–7.37 (5H, m), 8.73 (3H, br s); ^13^C NMR (100 MHz, CDCl_3_): *δ*/ppm 22.08, 28.93, 29.73, 40.36, 53.12, 53.32, 66.55, 128.05, 128.13, 128.55, 136.84, 156.72, 170.00. These spectral data are congruent with those previously reported [14, 15].

#### Synthesis of Boc-Phe-Lys(Cbz)-OMe (6b)

Boc-Phe (1.69 g, 6.4 mmol), PyBOP (3.76 g, 7.2 mmol) and diisopropylethylamine (2.00 mL, 12 mmol) were successively added to a solution of H-Lys(Cbz)-OMe hydrochloride (1.67 g, 5.1 mmol) in DMF (25 mL). The reaction mixture was then stirred for 24 h at room temperature. The mixture was poured into H_2_O (50 mL), and the organic materials were extracted with ethyl acetate (50 mL). The organic layer was successively washed with 1M KHSO_4_ (150 mL), saturated Na_2_CO_3_ aq. (150 mL), and brine (150 mL), and dried over anhydrous Na_2_SO_4_. After removing the solvent under reduced pressure, the crude product was purified by column chromatography on silica gel (140 g) with hexane/ethyl acetate = 1/2 as the eluent to give **6b** as a colorless solid (2.19 g, 80%). ^1^H NMR (400 MHz, CDCl_3_): *δ*/ppm 1.40 (9H, s), 1.48–1.56 (2H, m), 1.61–1.67 (3H, m), 1.78–1.83 (1H, m), 3.04 (2H, t, *J* = 6.9 Hz), 3.16 (2H, dd, *J* = 6.0, 13.6 Hz), 3.70 (3H, s), 4.35 (1H, dd, *J* = 7.5, 13.3 Hz), 4.54 (1H, dt, *J* = 5.2, 6.9 Hz), 4.94 (1H, br s), 5.09 (1H, t, 13.6 Hz), 5.10 (2H, s), 6.47 (1H, d, *J* = 7.5 Hz), 7.17–7.24 (5H, m), 7.27–7.36 (5H, m). ^13^C NMR (100 MHz, CDCl_3_): *δ*/ppm 22.17, 28.40, 29.29, 32.09, 38.19, 40.65, 52.01, 52.53, 55.90, 66.81, 77.37, 127.06, 128.24, 128.27, 128.66, 128.77, 129.47, 136.73(×2), 156.64(×2), 171.32, 172.30. These spectral data are congruent with those previously reported [16].

#### Synthesis of H-Phe-Orn(Cbz)-OMe hydrochloride

To a solution of **6a** (966 mg, 1.8 mmol) in 1,4-dioxane (20 mL), 4 M HCl in 1,4-dioxane (10mL) was added at room temperature, and the mixture was stirred overnight at room temperature. After the completion of the reaction, HCl and 1,4-dioxane were removed by evaporation. Then, the residual solvents were further removed by azeotropy with benzene three times to give H-Phe-Orn(Cbz)-OMe hydrochloride as a colorless solid (875 mg, quant.). ^1^H NMR (400 MHz, CDCl_3_): *δ*/ppm 1.25–1.63 (2H, m), 1.68–1.81 (2H, m), 3.09 (2H, br s), 3.26–3.34 (2H, m), 3.60 (3H, s), 4.37–4.45 (1H, m), 4.58–4.60 (1H, m), 5.00 (2H, s), 5.62 (1H, br s), 6.47 (1H, br s), 7.15–7.42 (10H, m), 8.32 (3H, br s); ^13^C NMR (100 MHz, DMSO-*d*_6_): *δ*/ppm 25.75, 28.17, 36.80, 51.91, 52.03, 53.17, 64.93, 65.15, 127.11, 127.73, 127.78, 128.37, 128.46, 129.60, 134.87, 137.24, 156.14, 168.18, 171.72. HR-ESIMS Calcd for C_23_H_30_N_3_O_5_ [M+H]^+^ 428.2180. Found 428.2134. This material was used directly in the next step without further purification.

#### Synthesis of Fmoc-Gly-Phe-Orn(Cbz)-OMe (7a)

Fmoc-Gly-OH (614 mg, 2.1 mmol), PyBOP (1.21 g, 2.3 mmol) in diisopropylethylamine (700 μL, 4 mmol) were added successively to a solution of H-Phe-Orn(Cbz)-OMe hydrochloride (888 mg, 1.9 mmol), and DMF (20 mL). The reaction mixture was stirred for 24h at room temperature. The mixture was poured into H_2_O (30 mL), and the organic materials were extracted with ethyl acetate (30 mL). The organic layer was successively washed with 1M KHSO_4_ (90 mL), saturated Na_2_CO_3_ aq. (90 mL), and brine (90 mL), and dried over anhydrous Na_2_SO_4_. After removal of the solvent under reduced pressure, the crude product was purified by column chromatography on silica gel (130 g) and eluted with hexane/ethyl acetate = 1/19 to give **7a** as a colorless solid (903 mg, 67%). ^1^H NMR (400 MHz, CDCl_3_): *δ*/ppm 1.41–1.49 (1H, m), 1.60–1.66 (2H, m), 1.80–1.89 (1H, m), 3.01–3.13 (4H, m), 3.69 (3H, s), 3.73–3.84 (2H, m), 4.16 (1H, t, *J* = 7.2 Hz), 4.39 (2H, d, *J* = 7.2 Hz), 4.51–4.56 (1H, m), 4.66–4.72 (1H, m), 5.04 (2H, s), 5.16 (1H, br s), 5.59 (1H, br s), 6.64–6.77 (2H, m), 7.15–7.32 (14H, m), 7.57 (2H, d, *J* = 7.0 Hz), 7.76 (2H, d, *J* = 7.0 Hz). ^13^C NMR (100 MHz, CDCl_3_): *δ*/ppm 25.83, 29.00, 37.56, 40.53, 44.62, 47.20, 52.13, 52.62, 54.44, 66.88, 67.39, 120.16, 125.15, 127.19, 127.25, 127.92, 128.22, 128.28, 128.67, 128.81, 129.38, 136.35, 136.60, 141.44, 143.84, 156.81(×2), 169.46, 170.50, 172.03. HR-ESIMS Calcd for C_40_H_42_N_4_NaO_8_ [M+Na]^+^ 729.2895. Found 729.2877.

#### Synthesis of H-Phe-Lys(Cbz)-OMe hydrochloride

To a solution of **6b** (1.65 g, 3 mmol) in 1,4-dioxane (10 mL), 4 M HCl in 1,4-dioxane (10mL) was added at room temperature, and the mixture was stirred at room temperature overnight. After the completion of the reaction, HCl and 1,4-dioxane were removed by evaporation. The residual solvents were further removed by azeotropy with benzene three times to give H-Phe-Lys(Cbz)-OMe hydrochloride as a colorless solid (1.51 g, quant.). ^1^H NMR (400 MHz, CDCl_3_): *δ*/ppm 1.22–1.27 (2H, m), 1.38–1.45 (2H, m), 1.61–1.64 (1H, m), 1.74–1.79 (1H, m), 3.09 (2H, br. s), 3.15 (2H, d, *J* = 7.1 Hz), 3.66 (3H, s), 4.31–4.42 (2H, m), 5.02 (2H, s), 6.03 (1H, br s), 7.15–7.22 (5H, m), 7.28–7.35 (5H, m); ^13^C NMR (100 MHz, CDCl_3_): *δ*/ppm 22.49, 28.93, 30.46, 36.67, 51.96, 52.18, 53.12, 64.93, 65.10, 127.02, 127.69, 127.73, 128.35, 128.38, 129.66, 134.94, 137.29, 156.10, 168.15, 171.80. HR-ESIMS Calcd for C_24_H_32_N_3_O_5_ [M+H]^+^ 442.2336. Found 442.2364. This material was used directly in the next step without further purification.

#### Synthesis of Fmoc-Gly-Phe-Lys(Cbz)-OMe (7b)

Fmoc-Gly-OH (633 mg, 2.1 mmol), PyBOP (1.23 g, 2.4 mmol) and diisopropylethylamine (1.1 mL, 6.3 mmol) were successively added to a solution of H-Phe-Lys(Cbz)-OMe hydrochloride (848 mg, 1.8 mmol) in DMF (8 mL). The reaction mixture was stirred for 24h at room temperature. The mixture was poured into H_2_O (20 mL), and the organic materials were extracted with ethyl acetate (20 mL). The organic layer was successively washed with 1M KHSO_4_ (60 mL), saturated Na_2_CO_3_ aq. (60 mL), and brine (60 mL), and dried over anhydrous Na_2_SO_4_. After removing the solvent under reduced pressure, the crude product was purified by column chromatography on silica gel (70 g) and eluted with CHCl_3_/MeOH = 49/1 to give **7b** as a colorless solid (720 mg, 56%). ^1^H NMR (400 MHz, CDCl_3_): *δ*/ppm 1.24–1.30 (2H, m), 1.42–1.48 (2H, m), 1.55–1.58 (1H, m), 1.79–1.82 (1H, m), 3.05–3.13 (4H, m), 3.68 (3H, s), 3.71–3.78 (2H, m), 4.18 (1H, t, *J* = 6.0 Hz), 4.39 (2H, d, *J* = 6.0 Hz) 4.47–4.52 (1H, m), 4.60–4.66 (1H, m), 5.06 (2H, s), 5.10 (1H, s), 5.47 (1H, br s), 6.48 (1H, br s), 7.165–7.32 (14H, m), 7.58 (2H, d, *J* = 7.5 Hz), 7.76 (2H, d, *J* = 7.5 Hz). ^13^C NMR (100 MHz, CDCl_3_): *δ*/ppm 22.27, 29.25, 31.57, 37.84, 40.55, 44.50, 47.14, 52.16, 52.45, 54.53, 66.76, 67.34, 120.08, 125.12, 127.08, 127.18, 127.84, 128.19(×2), 128.60, 128.70, 129.32, 136.63, 141.36, 143.82, 143.83, 156.72, 169.39, 170.63, 172.22. HR-ESIMS Calcd for C_41_H_44_N_4_NaO_8_ [M+Na]^+^ 743.3057. Found 743.3038.

#### Synthesis of H-Gly-Phe-Orn(Cbz)-OMe (8a)

Piperidine (400 μL, 4.1 mmol) was added to a solution of compound **7a** (802 mg, 1.1 mmol) in DMF (4.0 mL), and then the reaction mixture was stirred for 2 h at room temperature. After removal of the solvent under reduced pressure, the residue was purified by column chromatography on silica gel (60 g) and eluted with CHCl_3_/MeOH (9/1) to give **8a** as a colorless solid (506 mg, 92%). ^1^H NMR (400 MHz, CDCl_3_): *δ*/ppm 1.44–1.48 (2H, m), 1.63–1.66 (1H, m), 1.83–1.86 (1H, m), 3.01–3.30 (6H, m), 3.70 (3H, s), 4.51 (1H, dd, *J* = 2.2, 7.5 Hz), 4.66 (1H, dt, *J* = 14.6, 7.5 Hz), 5.07 (2H, s), 5.14–5.16 (1H, m), 6.88 (1H, d, *J* = 7.5 Hz), 7.19–7.35 (10H, m), 7.75 (1H, d, *J* = 7.5 Hz). ^13^C NMR (100 MHz, CDCl_3_): *δ*/ppm 25.75, 29.29, 37.75, 40.48, 44.66, 52.08, 52.58, 54.35, 66.77, 127.06, 128.23, 128.72, 129.38(×2), 136.72(×2), 156.68, 170.98, 172.16, 173.51. HR-ESIMS Calcd for C_25_H_32_N_4_NaO_6_ [M+Na]^+^ 507.2214. Found 507.2179.

#### Synthesis of H-Gly-Phe-Lys(Cbz)-OMe (8b)

Piperidine (2 mL, 20.3 mmol) was added to a solution of compound **7b** (1.78 g, 2.5 mmol) in DMF (8.0 mL), and the reaction mixture was stirred for 2 h at room temperature. After removing the solvent under reduced pressure, the residue was purified by column chromatography on silica gel (100 g) and eluted with dichloromethane/MeOH = 99/1 to give **8b** as a colorless solid (874 mg, 71%). ^1^H NMR (400 MHz, CDCl_3_): *δ*/ppm 1.25–1.31 (2H, m), 1.44–1.50 (2H, m), 1.59–1.74 (1H, m), 1.76–1.85 (1H, m), 3.03 (1H, dd, *J* = 13.9, 7.7 Hz), 3.08–3.19 (3H, m), 3.19–3.30 (1H, m), 3.69 (3H, s), 4.50 (1H, dt, *J* = 12.7, 7.7 Hz), 4.64 (1H, q, *J* = 7.4 Hz), 5.07–5.12 (2H, m), 5.27–5.30 (1H, m), 6.84 (1H, d, *J* = 8.0 Hz), 7.12–7.35 (10H, m), 7.71 (1H, d, *J* = 7.8 Hz). ^13^C NMR (100 MHz, DMSO-*d*_6_): *δ*/ppm 22.70, 29.02, 30.51, 37.97, 40.05, 43.50, 51.86, 52.07, 53.20, 65.17, 126.34, 127.74, 127.76, 128.05, 128.37, 129.32, 137.31, 137.50, 156.16, 171.01, 171.26, 172.41. HR-ESIMS Calcd for C_26_H_35_N_4_O_6_ [M+H]^+^ 499.2551. Found 499.2525.

#### Synthesis of 7PC-Gly-Phe-Orn(Cbz)-OH (4a)

DBU (300 μL, 2 mol) was added to the slurry of 7MCP (100 mg, 450 μmol) in MeOH (1.5 mL). Compound **8a** (506 mg, 1 mmol) was added to the mixture, which was then stirred at room temperature for 3 h. After the completion of the reaction, 0.01 M ^*n*^Bu_4_NCl aq. was added dropwise to the reaction mixture and the crude mixture was purified by gel filtration (LH-20, 80 g) and eluted with 0.01 M ^*n*^Bu_4_NCl. The pure fractions were collected and acidified with 6M HCl to pH 2, followed by filtration to yield compound **4a** (75 mg, 25%). ^1^H NMR (400 MHz, DMSO-*d*_6_): *δ*/ppm 1.20–1.78 (4H, m), 2.55–2.84 (2H, m), 2.88–3.05 (4H, m), 3.79–4.00 (2H, m), 4.12–4.24 (1H, m), 4.54–4.67 (1H, m), 5.00 (2H, s), 7.19–7.35 (12H, m), 8.17–8.39 (2H, m), 8.80–8.88 (2H, m), 11.75 (1H, br s), 12.63 (1H, br s). ^13^C NMR (100 MHz, DMSO-*d*_6_): *δ*/ppm 26.10, 28.34, 37,63, 42.22, 51.84, 53.56, 65.14, 126.24, 127.70, 127.99, 128.33, 129.22, 129.27, 131.86, 136.42, 137.24, 137.47, 137.66, 147.16, 154.51, 156.08, 156.12, 160.26, 162.77, 167.92, 171.03, 173.31. HR-ESIMS Calcd for C_31_H_32_N_9_O_8_ [M–H]^–^ 658.2379. Found 658.2356.

#### Synthesis of 7PC-Gly-Phe-Lys(Cbz)-OH (4b)

DBU (300 μL, 2 mol) was added to the slurry of 7MCP (109 mg, 492 μmol) in MeOH (2.0 mL). Compound **8b** (515 mg, 1 mmol) was added to the mixture, which was then stirred at room temperature for 3 h. After the completion of the reaction, 0.01 M ^*n*^Bu_4_NCl aq. was added dropwise to the reaction mixture and the crude mixture was purified by gel filtration (LH-20, 80 g) eluted with 0.01 M ^*n*^Bu_4_NCl. The pure fractions were collected and acidified with 6M HCl to pH 2, followed by filtration to yield compound **4b** (45 mg, 13%). ^1^H NMR (400 MHz, DMSO-*d*_6_): *δ*/ppm 1.24–1.36 (2H, m), 1.36–1.45 (2H, m), 1.56–1.76 (2H, m), 2.77 (1H, dd, *J* = 4.1, 11.8 Hz), 2.95–3.06 (3H, m), 3.83 (1H, dd, *J* = 5.9, 16.7 Hz), 3.95 (1H, dd, *J* = 5.9, 16.7 Hz), 4.12–4.17 (1H, m), 4.58–4.64 (1H, m), 4.90–5.07 (3H, m), 7.22–7.33 (12H, m), 8.23 (1H, d, *J* = 8.5 Hz), 8.30 (1H, d, *J* = 7.6 Hz), 8.79–8.89 (2H, m), 11.64 (1H, br. s), 12.58 (1H, br s). ^13^C NMR (100 MHz, DMSO-*d*_6_): *δ*/ppm 22.81, 29.08, 30.67, 37.64, 42.23, 52.03, 53.59, 65.13, 126.25, 127.72, 127.99, 128.33, 129.23, 129.28, 131.86, 136.46, 137.26, 137.67, 147.16, 154.56, 156.08(×2), 162.78, 167.95, 171.04, 173.45. HR-ESIMS Calcd for C_32_H_34_N_9_O_8_ [M–H]^–^ 672.2536. Found 672.2553.

### Mass expression of ricin toxin A-chain (RTA)

The pET28a plasmid containing the His-RTA expression sequence was purchased from GenScript and transformed into *E*. *coli* BL21(DE3) cells using the calcium treatment method. The transformed strains were incubated overnight at 32°C on LB agar culture supplemented with kanamycin. The generated colonies were picked up and inoculated into 5 litres of LB medium, and then incubated at 32°C with shaking. After approximately 6 h, the growth of the bacteria was visually confirmed by turbidity, IPTG was added to the medium to induce expression, and the cultivation was continued at 32°C overnight. Bacteria were collected by centrifugation and ultrasonically crushed to obtain a crude extract. His-tagged proteins were collected using a TALON column, dialyzed against 10 mM sodium acetate buffer (pH 4.0), and concentrated using an ultrafiltration filter to obtain the target proteins. Crystallization of RTA was then performed by the hanging drop vapor diffusion method using the obtained RTA protein solution.

### X-ray crystallography

RTA crystals were grown at 23°C using the hanging drop method from 6.4–7.8 mg/mL protein and 11–12% PEG2000, 0.2 M Lithium sulfate, 0.1 M sodium acetate, pH 4.5. Crystals of RTA complex with synthetic ligands were obtained by soaking RTA crystals in a crystallization reservoir containing 3.0 mM of the compound.

Diffraction data of RTA-ligand complex crystals were collected at 95 K using PILATUS3 S 2M detector on the beamline BL-5A Photon Factory (PF, Tsukuba, Japan). The data set was processed and scaled using the XDS [17] and SCALA [18] from the CCP4 suite [19]. Processed data were phased by the molecular replacement method using Molrep [20] with the coordinates of RTA bound with *N*-(*N*-(pterin-7-yl)carbonylglycyl)-L-phenylalanine (PDBID: 4HUO) [6]. Model building and electron density map inspection were done using Coot [21]. Following model building, refinement was performed using Refmac5 [22]. Crystallographic data for the complexes are presented in Table 2. Coordinates of the refined model of **4a**/RTA and **4b**/RTA complexes were in the Protein data Bank with accession codes 7Y4K and 7Y4M, respectively.

**Table 2:** Crystallographic data^†^ and refinement statistics.

	4a/RTA	4b/RTA
PDBID	7Y4K	7Y4M
Space group	*P* 4_1_2_1_2	*P* 4_1_2_1_2
No. of crystals	1	1
Unit cell parameters		
*a* (Å)	67.64	67.75
*b* (Å)	67.64	67.75
*c* (Å)	141.08	141.32
Resolution range (Å)	48.82–1.70	48.90–1.45 (1.53–1.45)
Observations	462,557	760,105
Unique reflections	36,955	59,220
Average multiplicity	12.5 (10.5)	12.8 (12.7)
Completeness (%)	100 (100)	100 (100)
Mean *I*/s(*I*)	27.9 (3.3)	37.9 (4.1)
*R*_merge_ (%) ^*a*^	5.4 (74.9)	3.1 (64.4)
*R*_meas_ (%)	5.6 (0.788)	3.2 (67.0)
*R*_*pim*_ (%)	1.6 (24.1)	0.9 (18.7)
CC(1/2) (%)	100 (89.4)	100 (94.2)
Refinement statistics (Å)	48.82–1.70	48.90–1.45
*R*_factor_ (%)	20.69	22.17
*R*_free_ (%) ^*b*^	23.1	24.19
No. of atoms		
Protein	2060	2072
Water	252	273
Ligand	25	59
RMSD from ideality		
bond length (Å)	0.0076	0.0071
bond angles (deg)	1.1839	1.1497
Average B factors (Å^2^)		
Protein	25.91	25.65
Water	39.13	39.49
Ligand	29.33	29.46
Ramachandran plot		
favoured region (%)	98.8	98.9
allowed region (%)	1.2	0.8
outlier region (%)	0	0.4

^†^ Values in parentheses correspond to values of the highest resolution shell.

^*a*^ *R*_merge_ = S_hkl_ S_i_ | *I*_hkl,i_–<*I*_hkl_> | / S_hkl_ S_i_ *I*_hkl,i_, where *I* = observed intensity and <*I*> = average intensity for multiple measurements.

^*b*^ *R*_free_ was monitored with 5% of the reflection data excluded from the refinement.

### RTA inhibitory activity measurements

The RTA inhibitory activity of the synthesized inhibitors was evaluated by measuring the amount of firefly luciferase produced by the *in vitro* translation reaction using rabbit reticulocyte lysate with luciferase quantification reagents.

A 1.5 mL eppendorf tube was filled with 0.2 μL of Amino Acid Mixtures (1 mM, Promega), 0.2 μL of RNasin^®^ ribonuclease inhibitor solution (1 U/μL, Promega), 0.2 μL of Luciferase Control RNA (1 mg/mL, Promega), 1.0 μL of RTA solution (0.5 nM, 20 mM HEPES buffer, pH 7.5), 1.0 μL of inhibitor solution (prepared with a minimum volume of dimethylsulfoxide and 20 mM HEPES buffer, pH 7.5), 7.0 μL of Reticulocyte Lysate (Promega) and made up to 10.0 μL with purified water. After incubation in a warm bath at 30°C for 90 min, the reaction was stopped by freezing at –20°C for 15 min. To the resulting translation product, 70 μL of purified water was added to obtaine a total volume of 80 μL, and 20 μL of each product was dispensed into a white 96-well plate. Luminescence was analyzed using the Luciferase Quantification Reagent brightlite^TM^ Plus (Perkin Elmer) and a microplate reader (ARVO X4, Perkin Elmer). The mean bioluminescence of each sample was determined in triplicates. The inhibition ratio was calculated using the following equation;

$$\mathrm{inhibition}\;\mathrm{ratio}\;(\%)=[1‐({\mathrm{RLU}}_{\mathrm{RTA}\&\mathrm{inhibitor}}{-\mathrm{RLU}}_{\mathrm{inhibitor}})/({\mathrm{RLU}}_{\mathrm{RTA}}{-\mathrm{RLU}}_{\mathrm{control}})]x100,$$

where RLU_RTA&inhibitor_ is the luminescence amount in the presence of both RTA and an inhibitor, RLU_inhibitor_ is the luminescence amount in the presence of an inhibitor but the absence of RTA, RLU_RTA_ is the luminescence amount in the presence of RTA but the absence of an inhibitor, and RLU_control_ is the luminescence amount in the absence of RTA nor an inhibitor.

## Supporting information

S1 File Proton (^1^H) and carbon (^13^C) NMR spectra of new compounds. (PDF)

S1 Dataset Raw NMR data. (ZIP)
