# Correction: Pterin-based small molecule inhibitor capable of binding to the secondary pocket in the active site of ricin-toxin A chain

**DOI:** 10.1371/journal.pone.0304251

**Published:** 2024-05-17

**Authors:** Ryota Saito, Masaru Goto, Shun Katakura, Taro Ohba, Rena Kawata, Kazuki Nagatsu, Shoko Higashi, Kaede Kurisu, Kaori Matsumoto, Kouta Ohtsuka

In Fig 3, the Asn48 in the left side of the figure is incorrect. It should have been Asn78. Please see the correct Fig 3 here.

**Fig 3:**
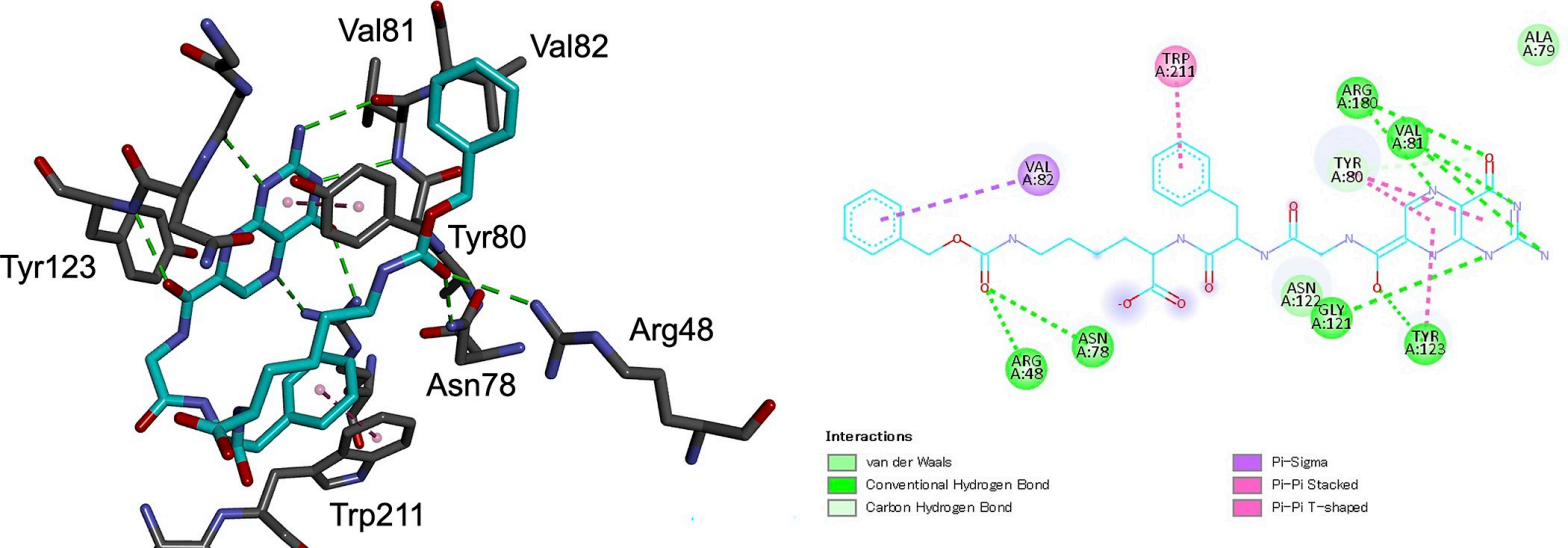
Interactions between 4b and RTA.

All interactions were detected and visualized by Discovery Studio Visualizer [11] in both 2D (right) and 3D (left) styles.

Consequently, in the Results and Discussion, the ninth sentence of the second paragraph is incorrect. The correct sentence is: Besides, the carbonyl group of the Cbz in 4b made hydrogen bonds with Arg48 and Asn78 in the “entrance” of the secondary pocket at 3.15 Å and 2.67 Å, respectively, and accordingly the benzene ring of Cbz group was placed in the secondary pocket (Fig 3).

Moreover, in Table 1, the IC50 values for compounds 4a and 4b are swapped. The correct IC50 value for 4a should be 485±33 μM, and 33.4±2.7 μM for 4b. Please see the correct Table 1 here.

**Table 1:** RTA inhibitory activity of 1, 4a, and 4b.

Compound	IC_50_ /μM
**1**	30.8 ± 9.0
**4a**	485 ± 33
**4b**	33.4 ± 2.7
