# Supplementary material for: Pterin-based small molecule inhibitor capable of binding to the secondary pocket in the active site of ricin-toxin A chain
Source: PLoS One. 2022 Dec 12;17(12):e0277770. doi: 10.1371/journal.pone.0277770 (PMC9744275; doi:10.1371/journal.pone.0277770)
Supplement: S1 File — (PDF) [file pone.0277770.s001.pdf]

Supporting Information

Pterin-based small molecule inhibitor capable of binding to the secondary pocket in the active site of ricin-toxin A chain

Ryota Saito,[1,2*] Masaru Goto,[3] Shun Katakura,[1] Taro Ohba,[3] Rena Kawata,[3] Kazuki Nagatsu,[3] Shoko Higashi,[1] Kaede Kurisu,[1] Kaori Matsumoto,[1] and Kouta Ohtsuka[1]

[1] Department of Chemistry, Faculty of Science, Toho University, Funabashi, Chiba 274-8510, Japan

[2] Research Center for Materials with Integrated Properties, Toho University, Funabashi, Chiba 274-8510, Japan

[3] Department of Molecular Bioscience, Faculty of Science, Toho University, Funabashi, Chiba 274-8510, Japan

Table of Contents

1. General Information p.1
2. NMR spectra
S1 Fig. ^{1}H-NMR ($CDCl_3$, 400 MHz) spectrum of Boc-Phe-Orn(Cbz)-OMe (**6a**) p.2
S2 Fig. ^{13}C-NMR ($CDCl_3$, 100 MHz) spectrum of Boc-Phe-Orn(Cbz)-OMe (**6a**) p.3
S3 Fig. ^{1}H-NMR ($CDCl_3$, 400 MHz) spectrum of Fmoc-Gly-Phe-Orn(Cbz)-OMe (**7a**) p.4
S4 Fig. ^{13}C-NMR ($CDCl_3$, 100 MHz) spectrum of Fmoc-Gly-Phe-Orn(Cbz)-OMe (**7a**) p.5
S5 Fig. ^{1}H-NMR ($CDCl_3$, 400 MHz) spectrum of Fmoc-Gly-Phe-Lys(Cbz)-OMe (**7b**) p.6
S6 Fig. ^{13}C-NMR ($CDCl_3$, 100 MHz) spectrum of Fmoc-Gly-Phe-Lys(Cbz)-OMe (**7b**) p.7
S7 Fig. ^{1}H-NMR ($CDCl_3$, 400 MHz) spectrum of H-Gly-Phe-Orn(Cbz)-OMe (**8a**) p.8
S8 Fig. ^{13}C-NMR ($CDCl_3$, 100 MHz) spectrum of H-Gly-Phe-Orn(Cbz)-OMe (**8a**) p.9
S9 Fig. ^{1}H-NMR ($CDCl_3$, 400 MHz) spectrum of H-Gly-Phe-Lys(Cbz)-OMe (**8b**) p.10
S10 Fig. ^{13}C-NMR ($CDCl_3$, 100 MHz) spectrum of H-Gly-Phe-Lys(Cbz)-OMe (**8b**) p.11
S11 Fig. ^{1}H-NMR (DMSO-d_6, 400 MHz) spectrum of 7PC-Gly-Phe-Orn(Cbz)-OH (**4a**) p.12
S12 Fig. ^{13}C-NMR (DMSO-d_6, 100 MH) spectrum of 7PC-Gly-Phe-Orn(Cbz)-OH (**4a**) p.13
S13 Fig. ^{1}H-NMR (DMSO-d_6, 400 MHz) spectrum of 7PC-Gly-Phe-Lys(Cbz)-OH (**4b**) p.14
S14 Fig. ^{13}C-NMR (DMSO-d_6, 100 MHz) spectrum of 7PC-Gly-Phe-Lys(Cbz)-OH (**4b**) p.15

1. General Information

^{1}H-NMR and ^{13}C-NMR spectra were recorded on an ECP-400 spectrometer (JEOL Ltd., Japan) or an Avance II 400 spectrometer (Bruker Biospin, Billerica, MA, USA). The raw NMR signals (FID) were multiplied with the exponential and the Gaussian window functions using a MestReNova (Mnova) NMR software ver. 14.2.1 (Mestrelab Research, Santiago de Compostela, Spain. https://mestrelab.com/). Chemical shifts (δ) are reported in ppm using tetramethylsilane or an undeuterated solvent as internal standards in the deuterated solvent used.

2. NMR spectra

S1 Fig. ^{1}H-NMR ($CDCl_3$, 400 MHz) spectrum of Boc-Phe-Orn(Cbz)-OMe (**6a**)

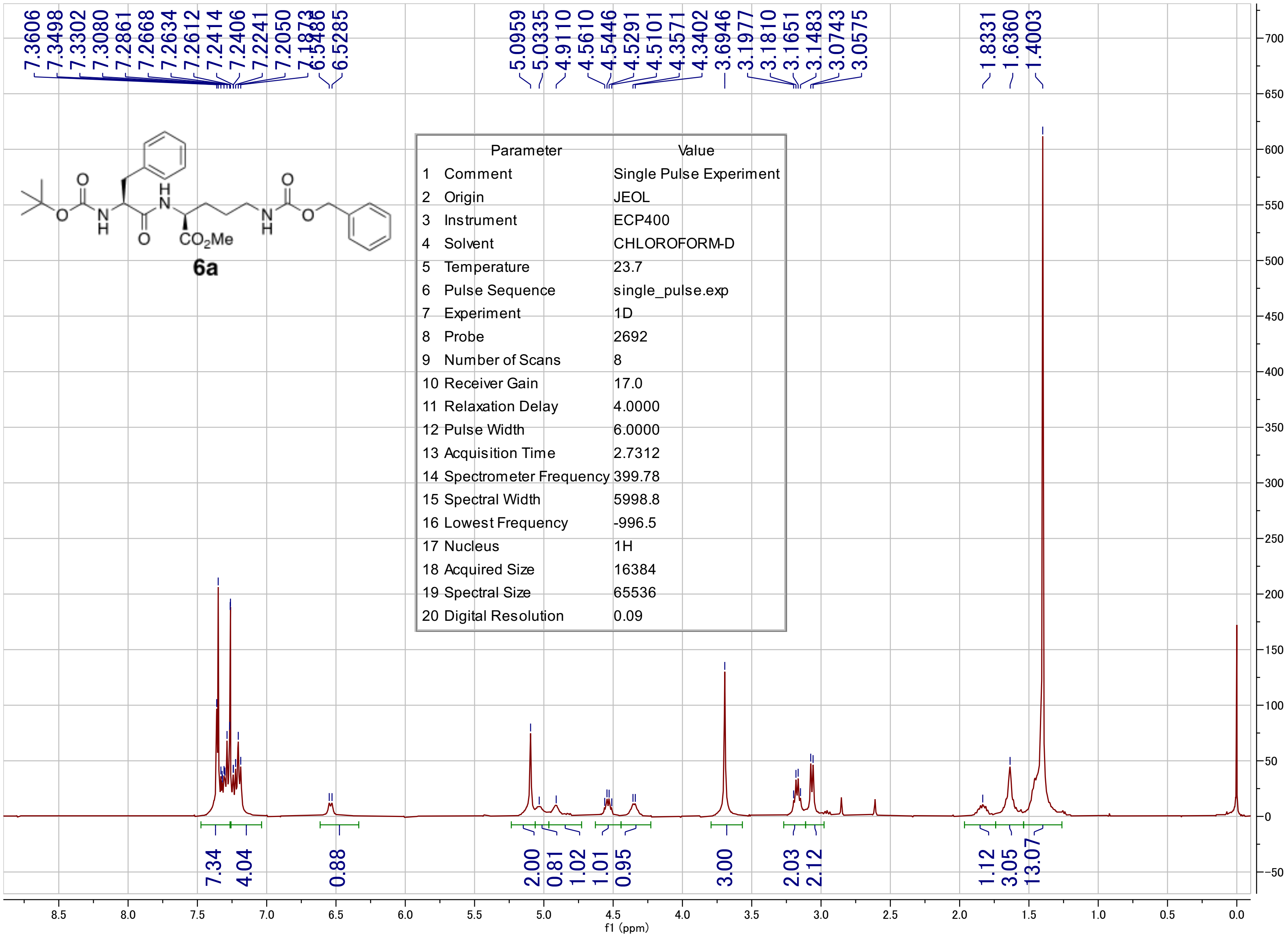

S2 Fig. ^{13}C-NMR ($CDCl_3$, 100 MHz) spectrum of Boc-Phe-Orn(Cbz)-OMe (**6a**)

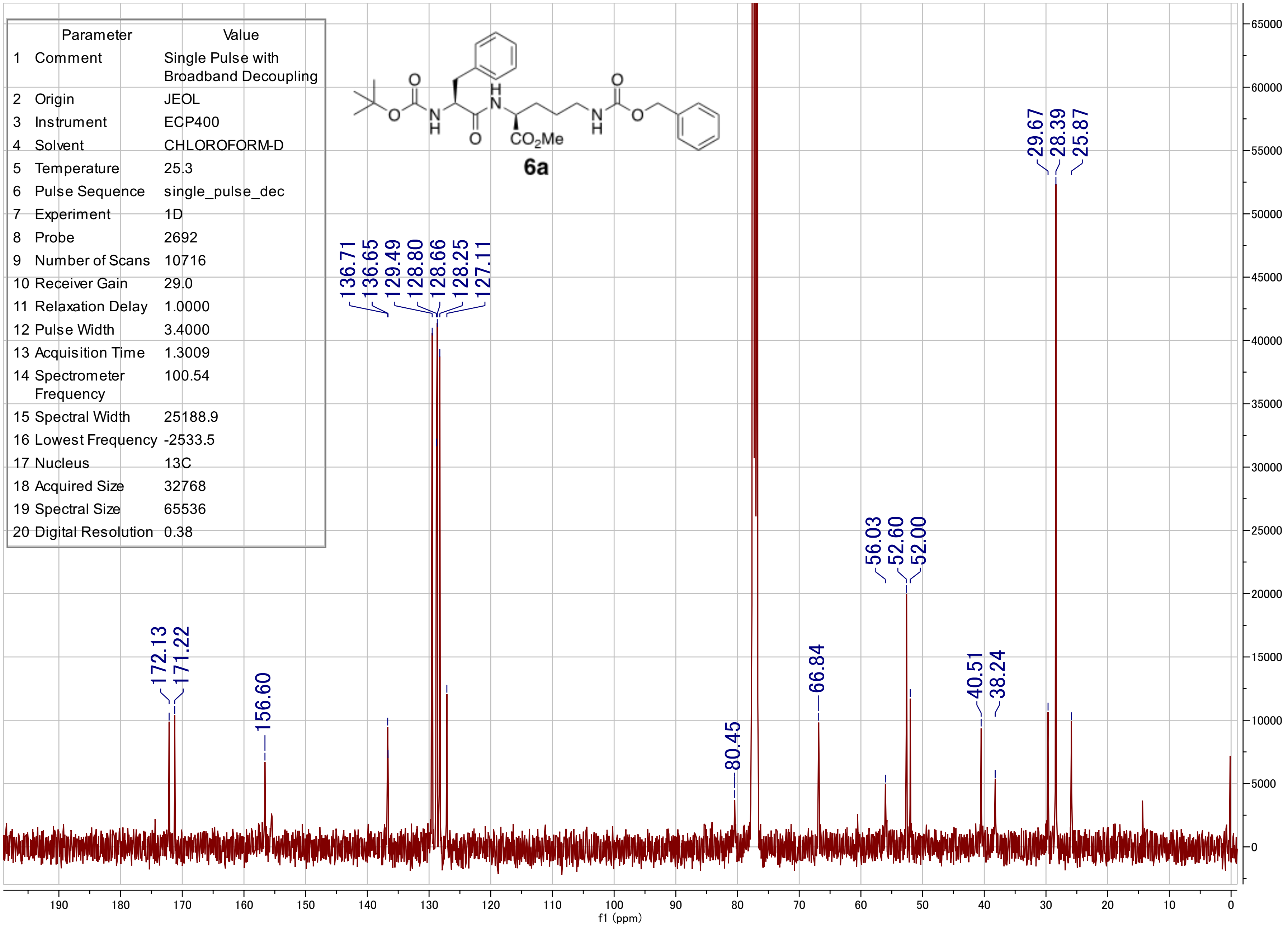

S3 Fig. ^{1}H-NMR ($CDCl_3$, 400 MHz) spectrum of Fmoc-Gly-Phe-Orn(Cbz)-OMe (**7a**)

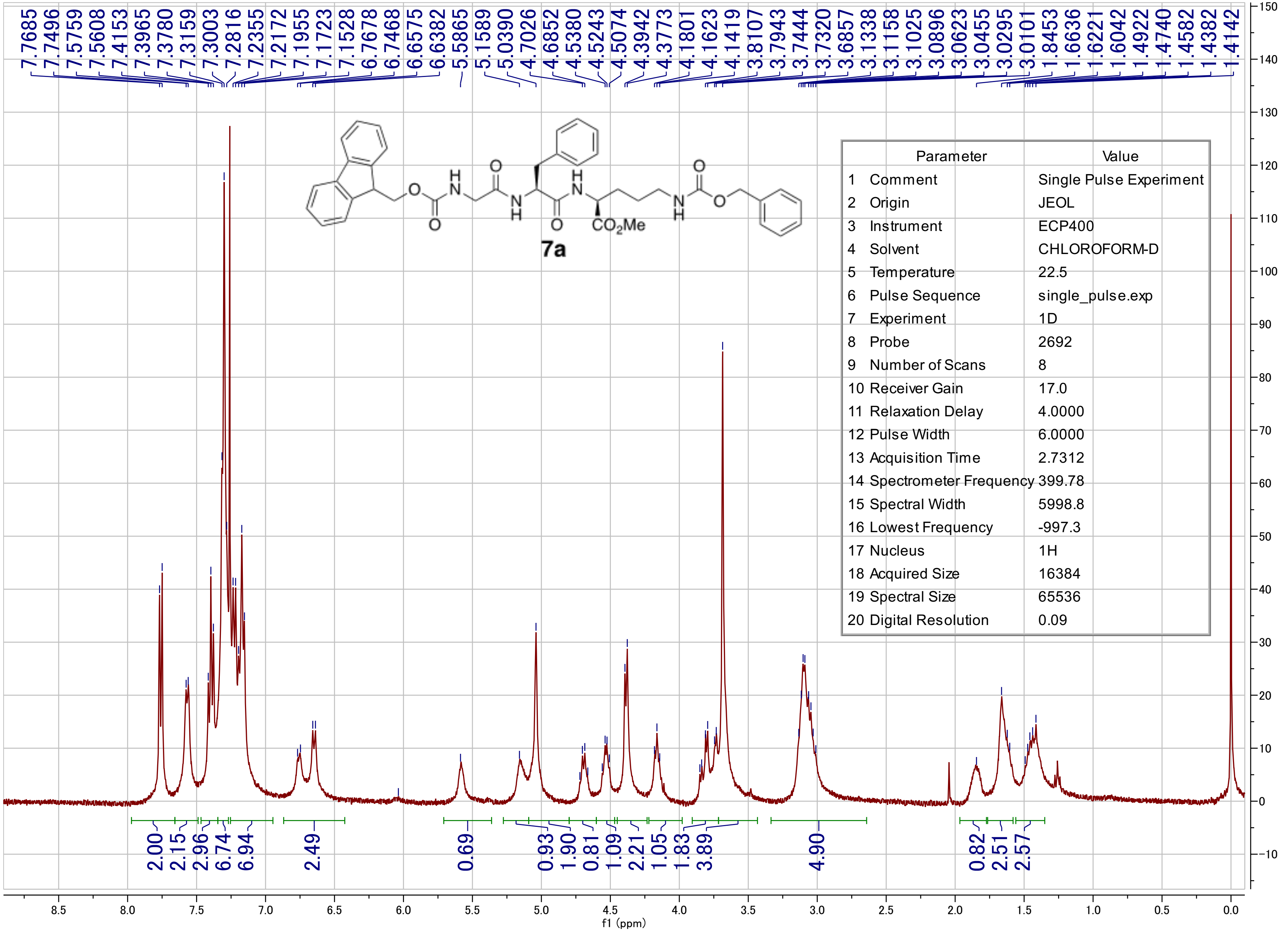

S4 Fig. ^{13}C-NMR ($CDCl_3$, 100 MHz) spectrum of Fmoc-Gly-Phe-Orn(Cbz)-OMe (**7a**)

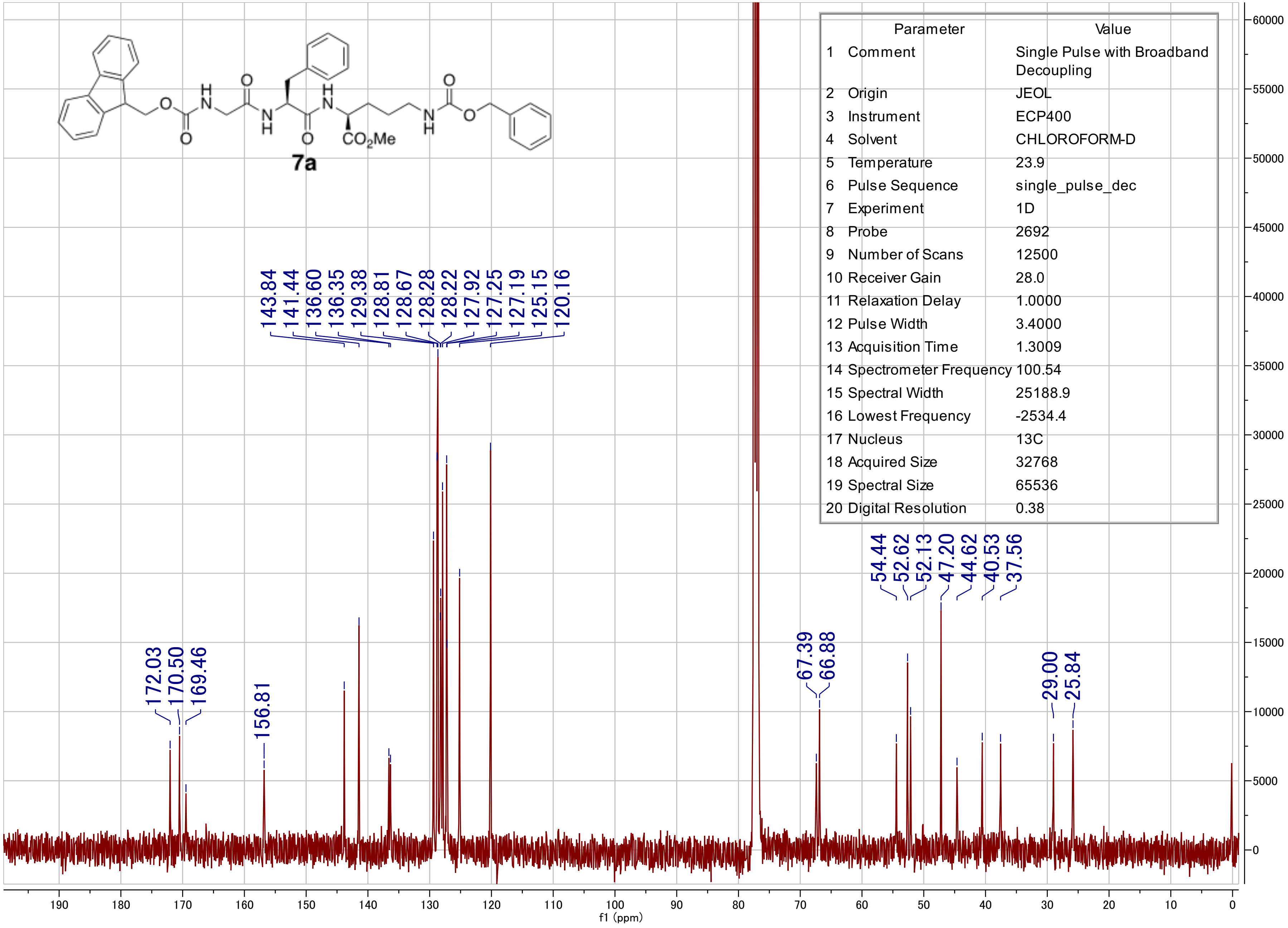

S5 Fig. ^{1}H-NMR ($CDCl_3$, 400 MHz) spectrum of Fmoc-Gly-Phe-Lys(Cbz)-OMe (**7b**)

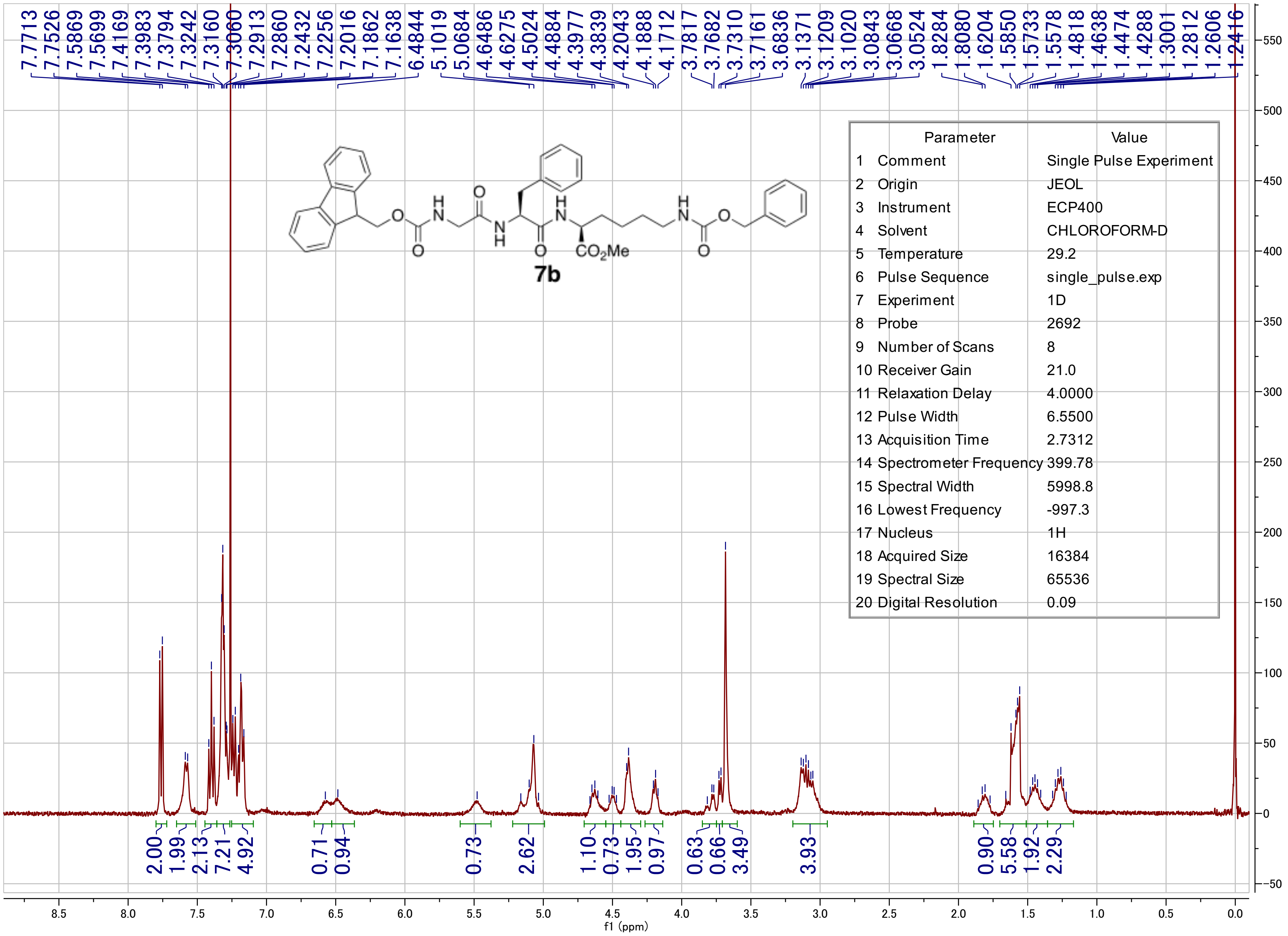

S6 Fig. ^{13}C-NMR ($CDCl_3$, 100 MHz) spectrum of Fmoc-Gly-Phe-Lys(Cbz)-OMe (**7b**)

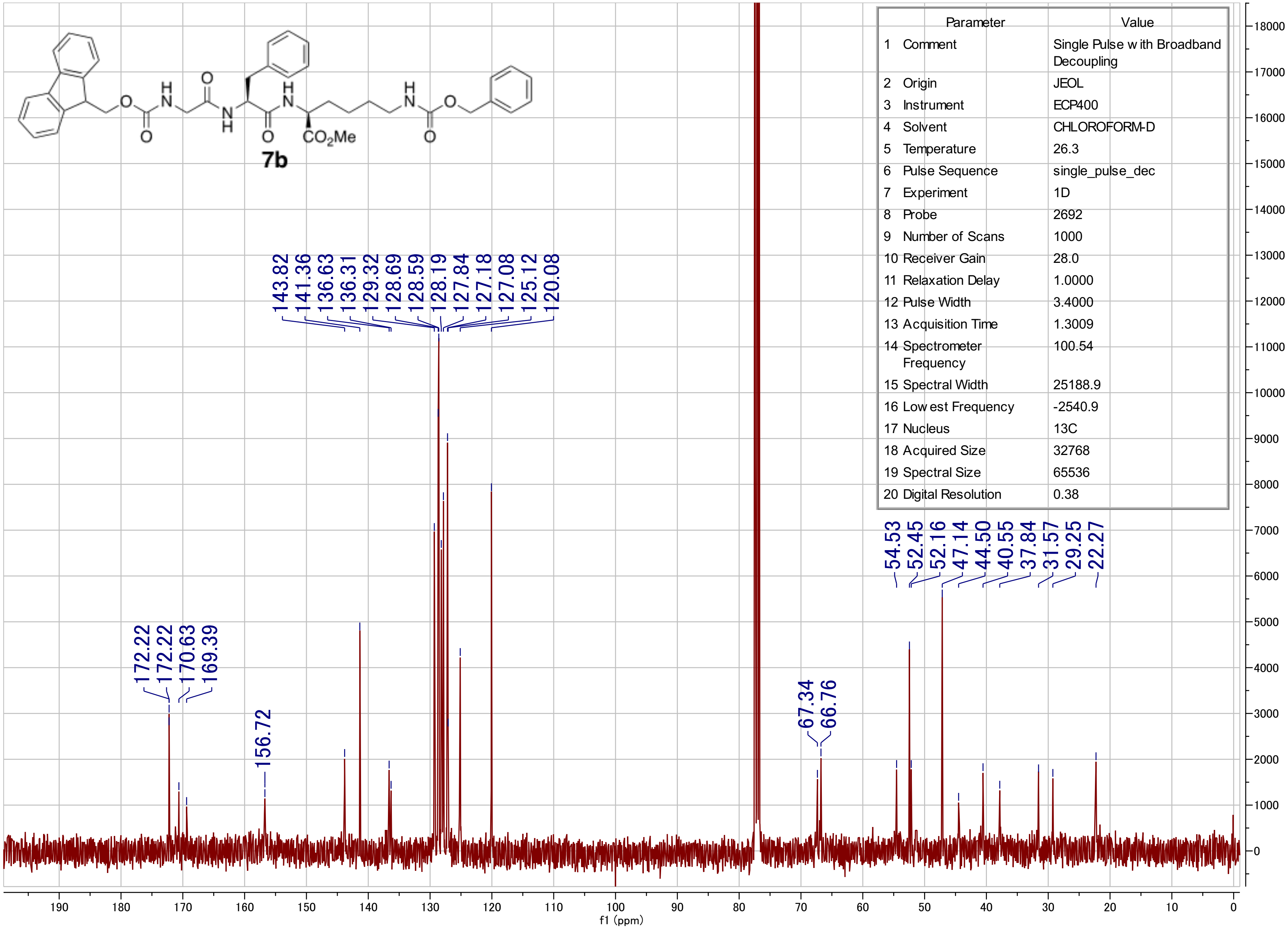

S7 Fig. ^{1}H-NMR ($CDCl_3$, 400 MHz) spectrum of H-Gly-Phe-Orn(Cbz)-OMe (**8a**)

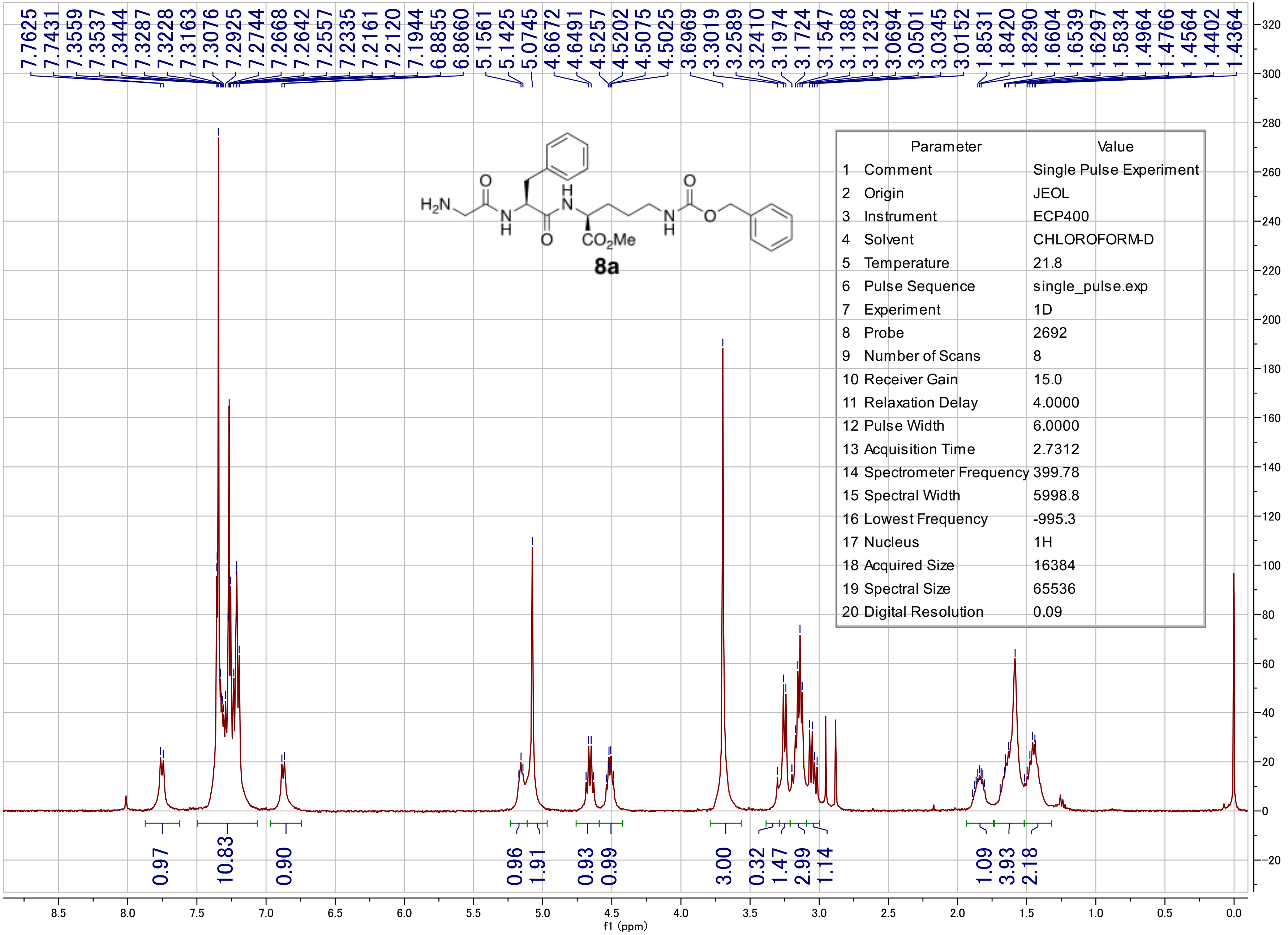

S8 Fig. ^{13}C-NMR ($CDCl_3$, 100 MHz) spectrum of H-Gly-Phe-Orn(Cbz)-OMe (**8a**)

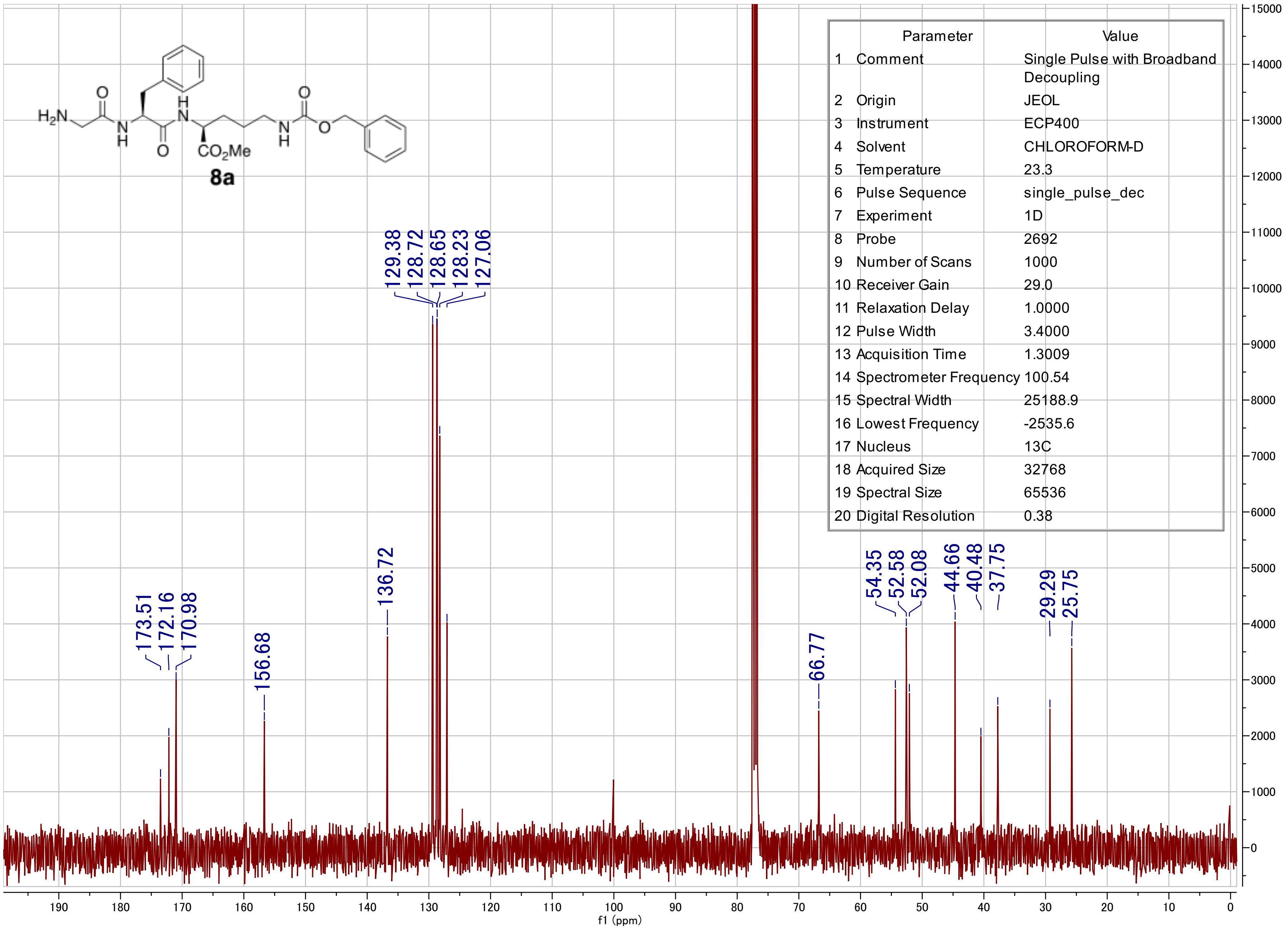

S9 Fig. ^{1}H-NMR ($CDCl_3$, 400 MHz) spectrum of H-Gly-Phe-Lys(Cbz)-OMe (**8b**)

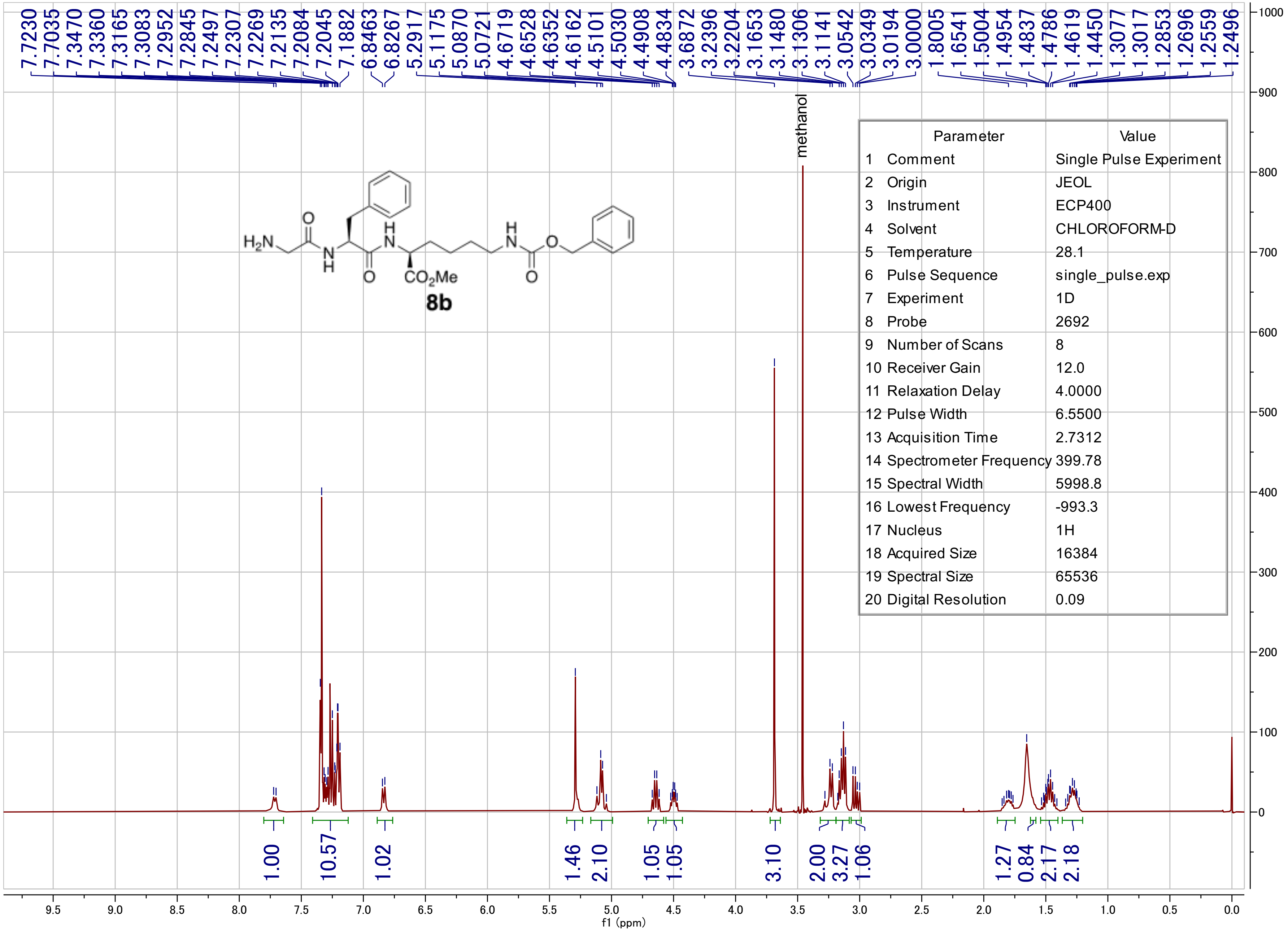

S10 Fig. ^{13}C-NMR (DMSO-d_6, 100 MHz) spectrum of H-Gly-Phe-Lys(Cbz)-OMe (**8b**)

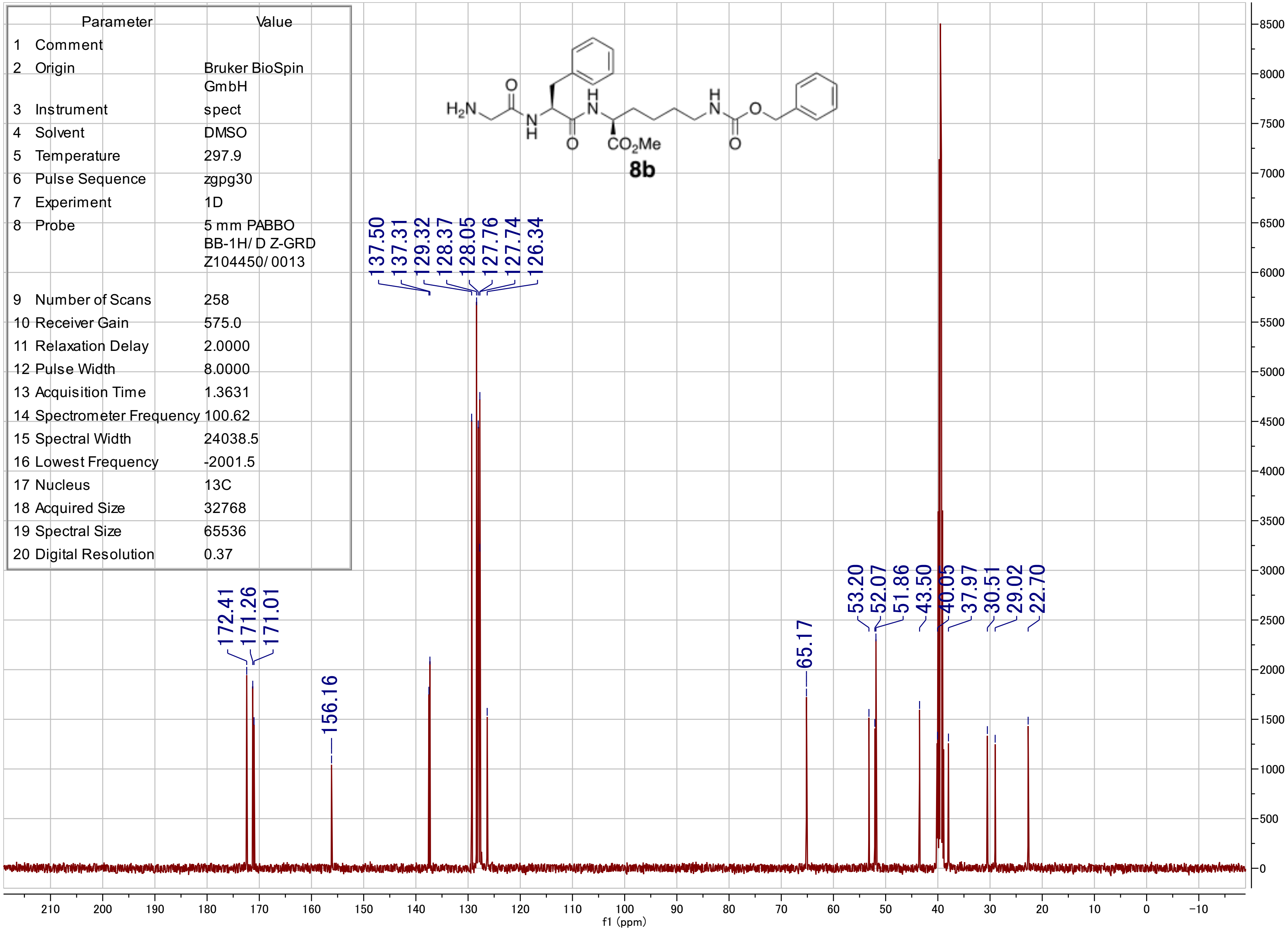

S11 Fig. ^{1}H-NMR (DMSO-d_6, 400 MHz) spectrum of 7PC-Gly-Phe-Orn(Cbz)-OH (**4a**)

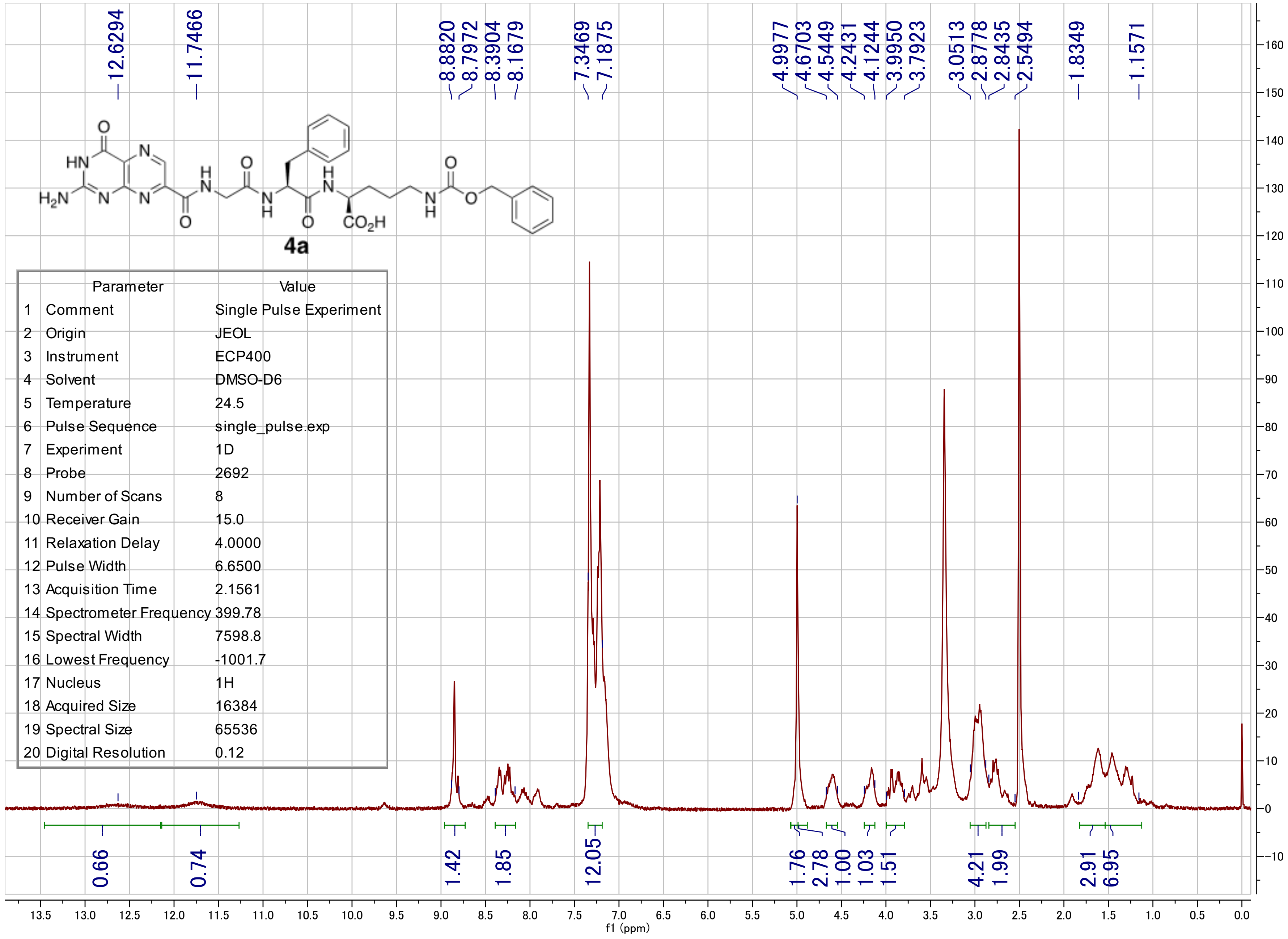

S12 Fig. ^{13}C-NMR (DMSO-d_6, 100 MHz) spectrum of 7PC-Gly-Phe-Orn(Cbz)-OH (**4a**)

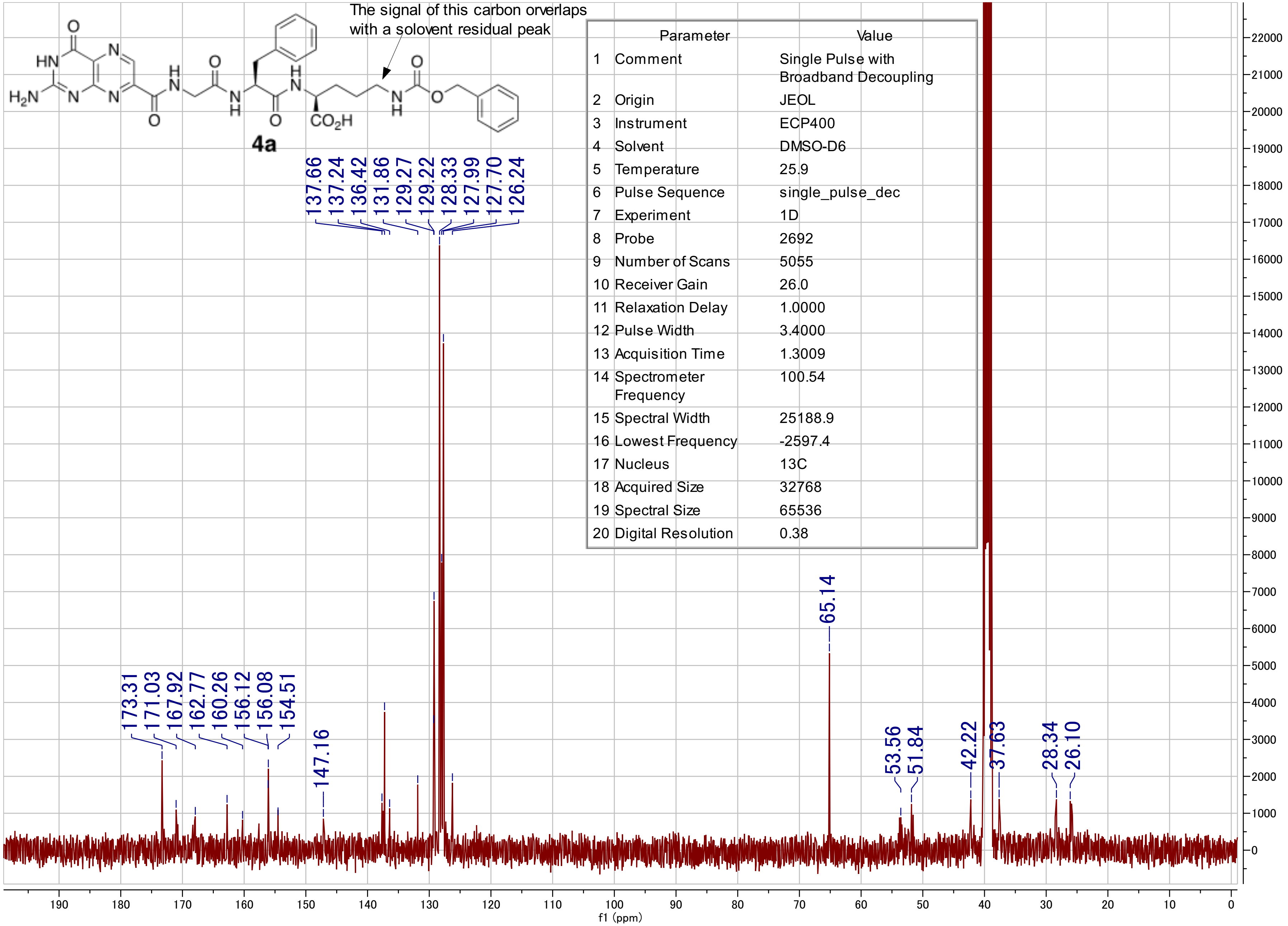

	Parameter	Value
1	Comment	Single Pulse with Broadband Decoupling
2	Origin	JEOL
3	Instrument	ECP400
4	Solvent	DMSO-D6
5	Temperature	25.9
6	Pulse Sequence	single_pulse_dec
7	Experiment	1D
8	Probe	2692
9	Number of Scans	5055
10	Receiver Gain	26.0
11	Relaxation Delay	1.0000
12	Pulse Width	3.4000
13	Acquisition Time	1.3009
14	Spectrometer Frequency	100.54
15	Spectral Width	25188.9
16	Lowest Frequency	-2597.4
17	Nucleus	13C
18	Acquired Size	32768
19	Spectral Size	65536
20	Digital Resolution	0.38

S13 Fig. ^{1}H-NMR (DMSO-d_6, 400 MHz) spectrum of 7PC-Gly-Phe-Lys(Cbz)-OH (**4b**)

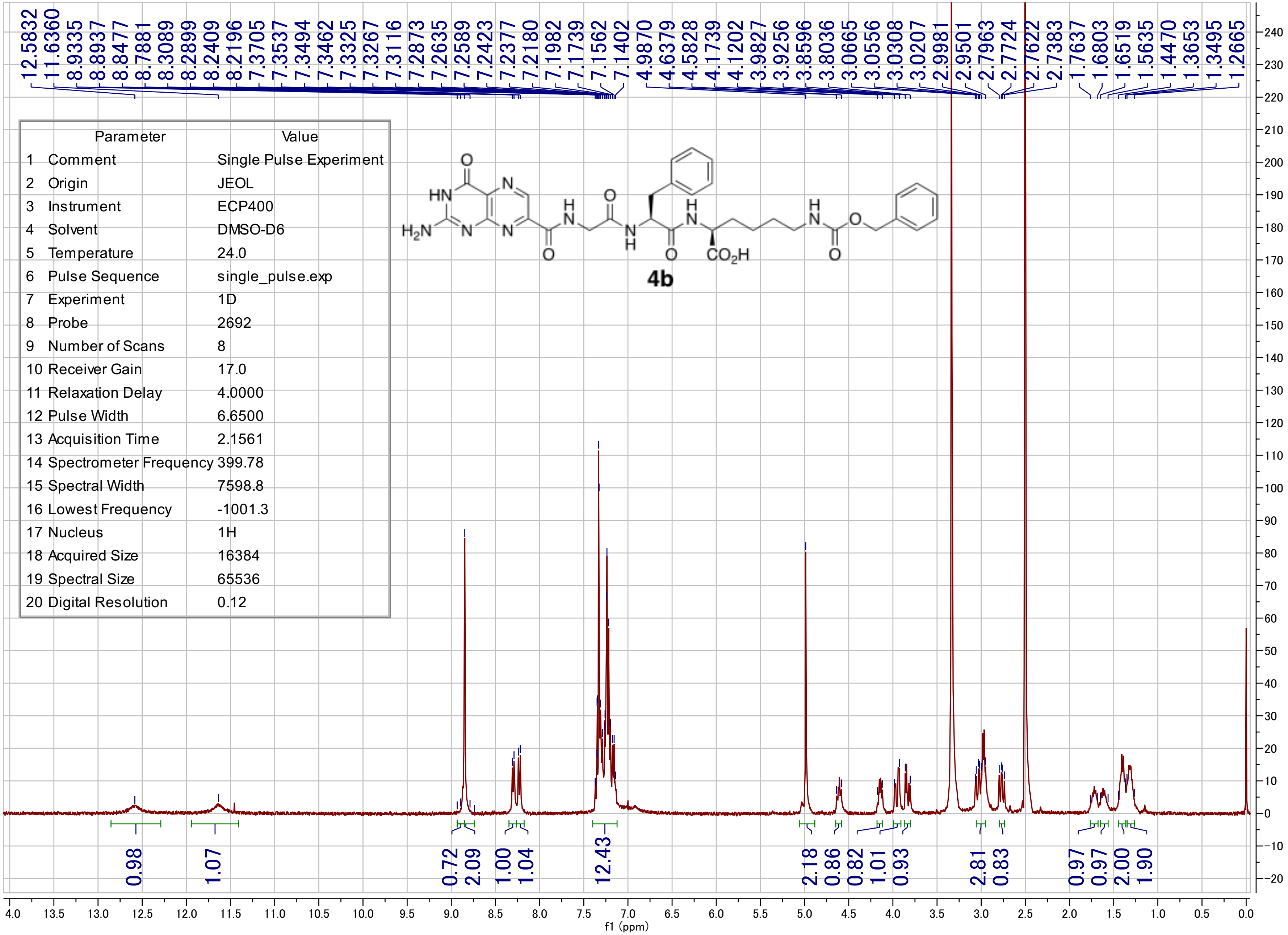

S14 Fig. ^{13}C-NMR (DMSO-d_6, 100 MHz) spectrum of 7PC-Gly-Phe-Lys(Cbz)-OH (**4b**)

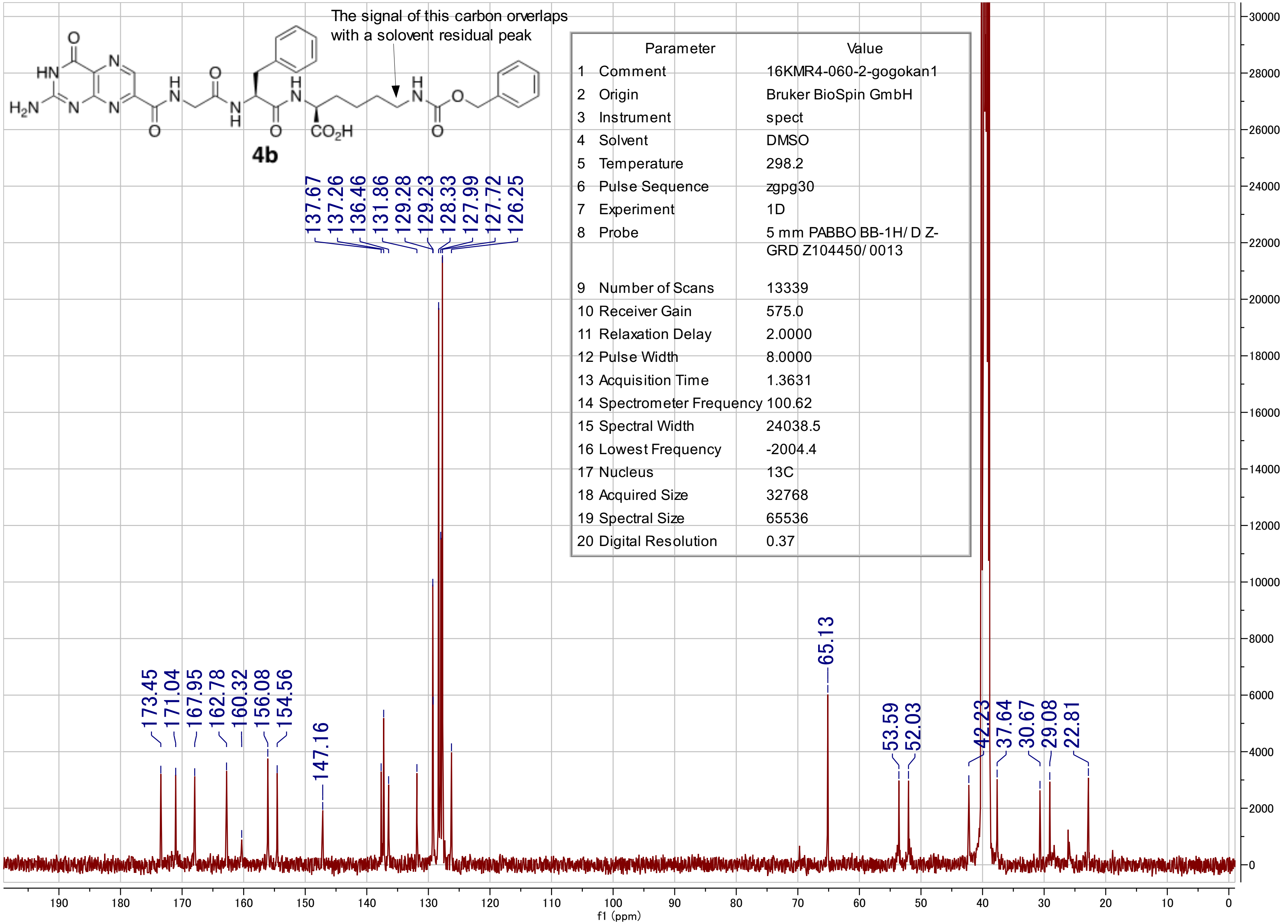

Parameter		Value
1	Comment	16KMR4-060-2-gogokan1
2	Origin	Bruker BioSpin GmbH
3	Instrument	spect
4	Solvent	DMSO
5	Temperature	298.2
6	Pulse Sequence	zgpg30
7	Experiment	1D
8	Probe	5 mm PABBO BB-1H/ D Z-GRD Z104450/ 0013
9	Number of Scans	13339
10	Receiver Gain	575.0
11	Relaxation Delay	2.0000
12	Pulse Width	8.0000
13	Acquisition Time	1.3631
14	Spectrometer Frequency	100.62
15	Spectral Width	24038.5
16	Lowest Frequency	-2004.4
17	Nucleus	13C
18	Acquired Size	32768
19	Spectral Size	65536
20	Digital Resolution	0.37
