# Supplementary figures and images for: Pterin-based small molecule inhibitor capable of binding to the secondary pocket in the active site of ricin-toxin A chain
Source: PLoS One. 2022 Dec 12;17(12):e0277770. doi: 10.1371/journal.pone.0277770 (PMC9744275; doi:10.1371/journal.pone.0277770)

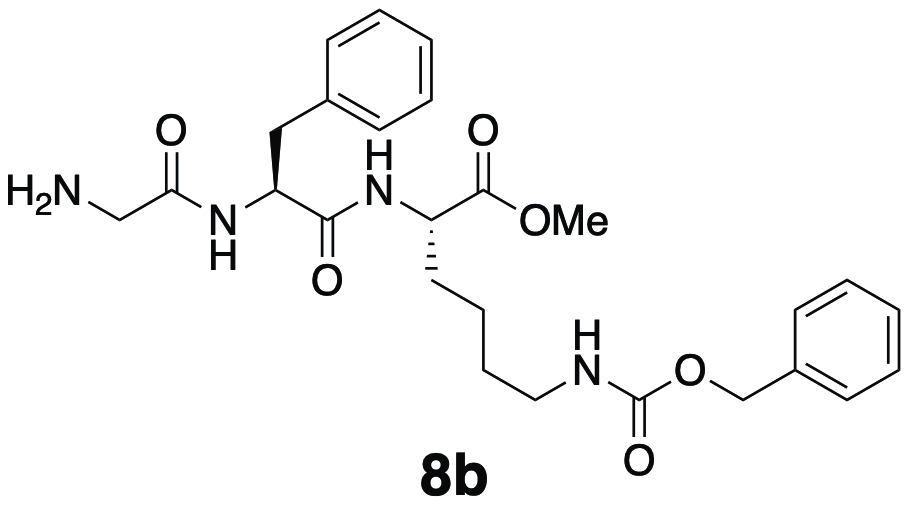

Supplement: S1 Dataset — (ZIP) [file pone.0277770.s002.zip › raw data/8b.tiff]

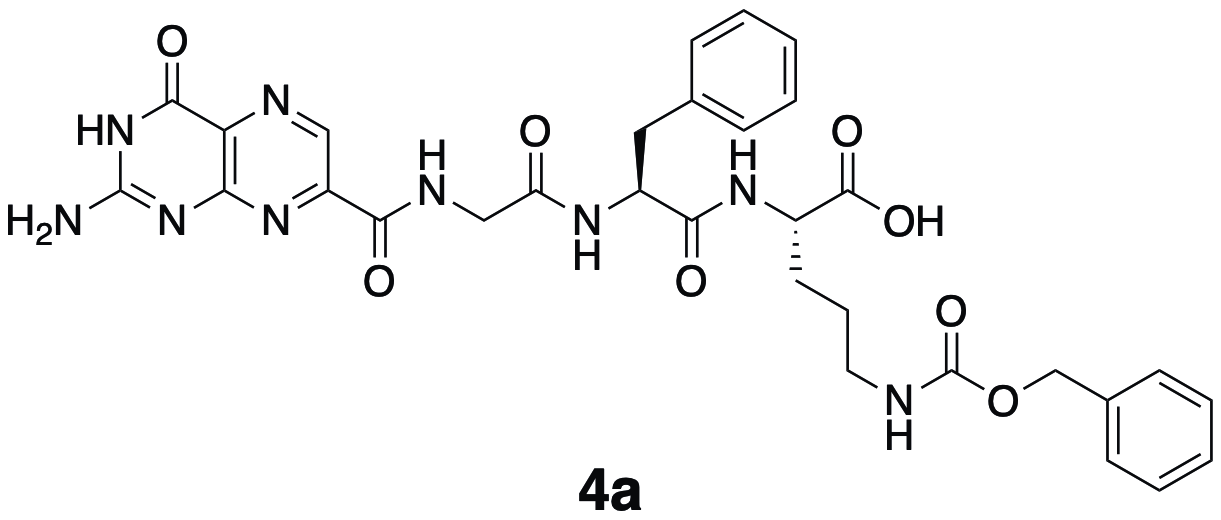

Supplement: S1 Dataset — (ZIP) [file pone.0277770.s002.zip › raw data/4a.tiff]

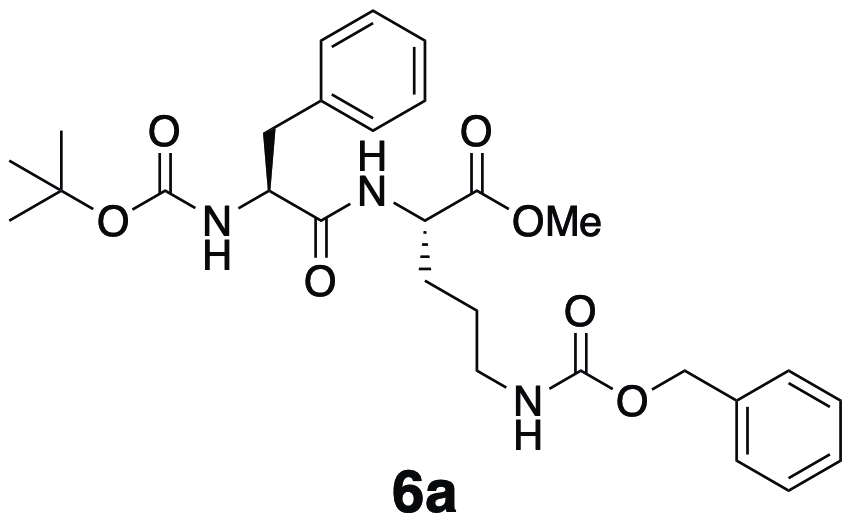

Supplement: S1 Dataset — (ZIP) [file pone.0277770.s002.zip › raw data/6a.tiff]

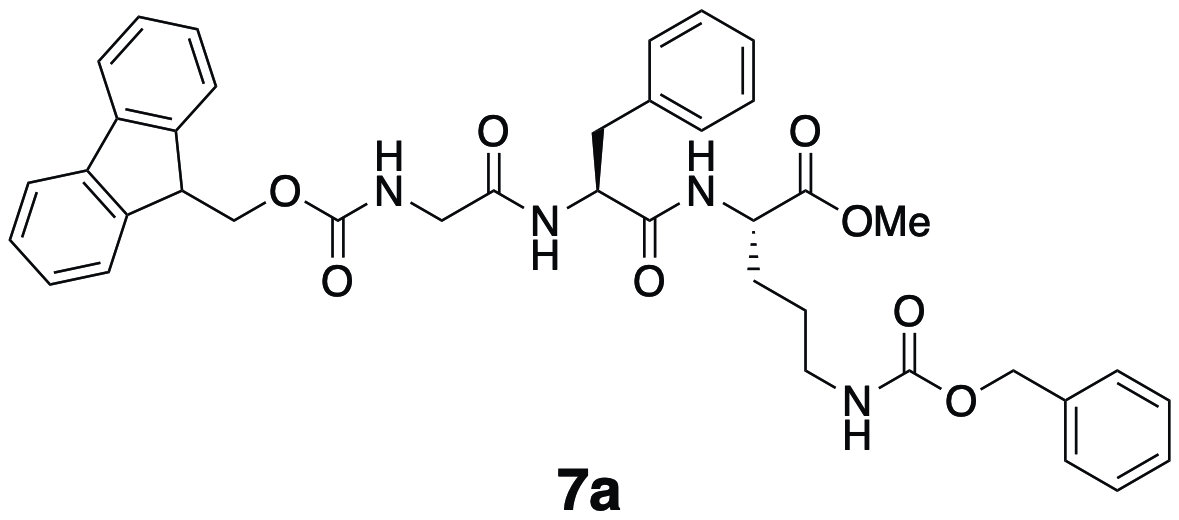

Supplement: S1 Dataset — (ZIP) [file pone.0277770.s002.zip › raw data/7a.tiff]

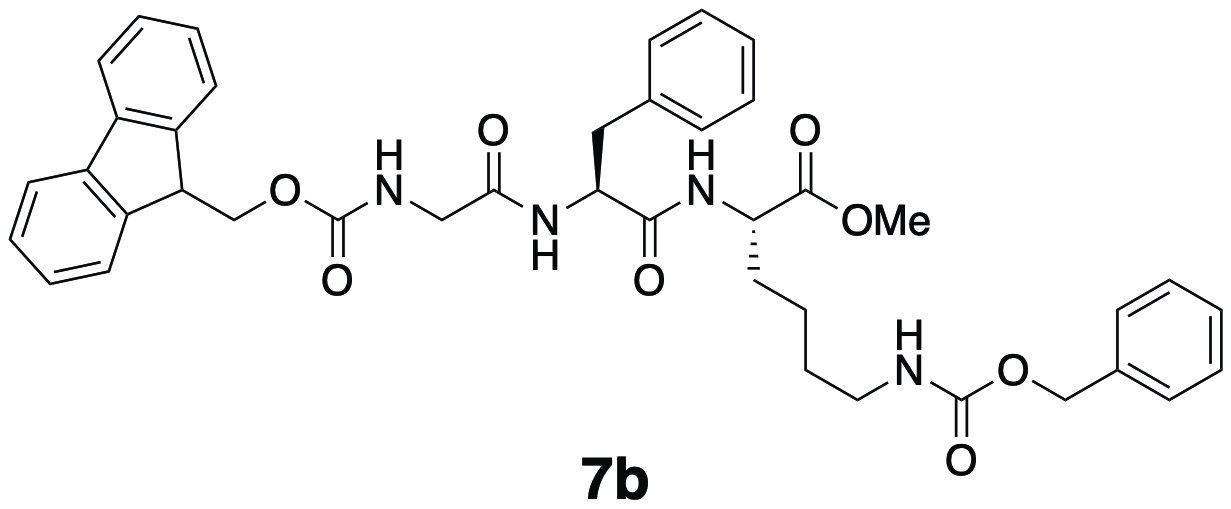

Supplement: S1 Dataset — (ZIP) [file pone.0277770.s002.zip › raw data/7b.tiff]

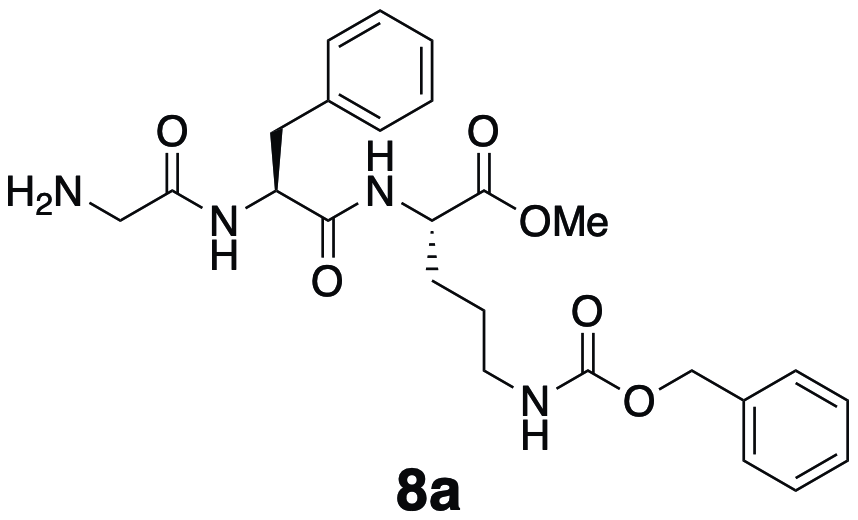

Supplement: S1 Dataset — (ZIP) [file pone.0277770.s002.zip › raw data/8a.tiff]

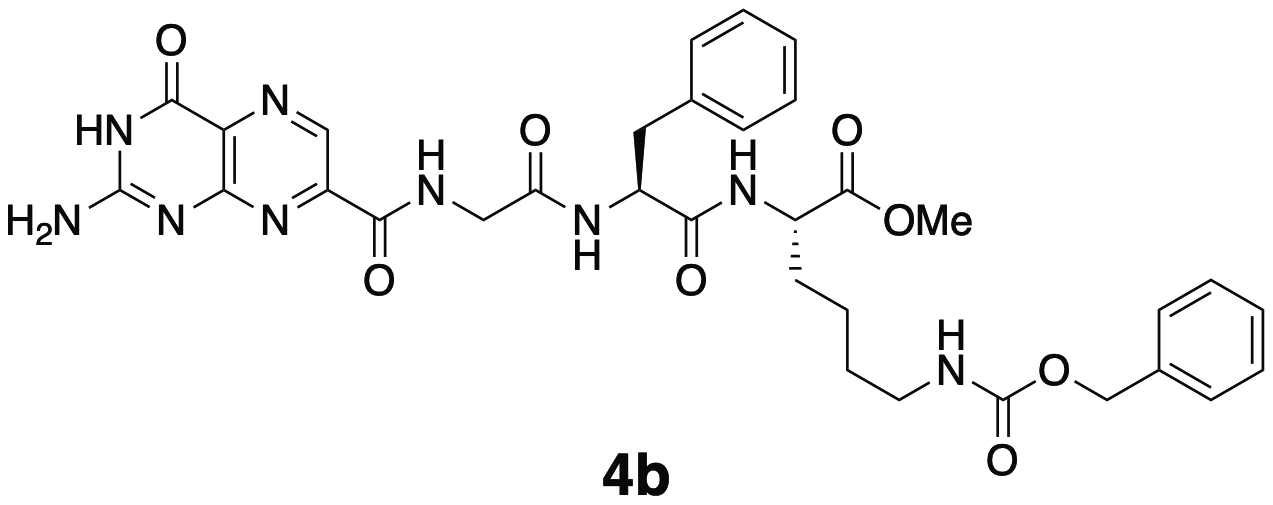

Supplement: S1 Dataset — (ZIP) [file pone.0277770.s002.zip › raw data/4b.tiff]
